# A cognitive synergetic hierarchical framework for UAV swarm combat via speculative inference and role-decoupled reinforcement learning

**DOI:** 10.3389/fnbot.2026.1799054

**Published:** 2026-05-19

**Authors:** Lixa Wang, Yunqing Liu, Linyang Guo, Ran Zhang, Yu Cui, Yihan Cui, Chunlin Xu

**Affiliations:** 1 School of Electronics and Information Engineering, Changchun University of Science and Technology, Changchun, Jilin, China; 2 Jiangsu Shuguang Electro-Optics Co., LTD., Yangzhou, Jiangsu, China; 3 Yangzhou University, Yangzhou, Jiangsu, China; 4 Army Arms University of PLA, Changchun, Jilin, China

**Keywords:** hierarchical reinforcement learning, large language models, speculative decoding, tactical decision-making, UAV swarm

## Abstract

In the high-stakes arena of aerial combat—a domain defined by extreme dynamics and unforgiving physical constraints—UAV swarms are currently squeezed between two extremes: the “tactical short-sightedness” of Multi-Agent Reinforcement Learning (MARL) and the “inference lag” of Large Language Models (LLMs). While MARL struggles to internalize the complex maneuverability priors required for expert flight, LLMs are simply too heavy to meet millisecond-level control demands. We bridge this gap by introducing a cognitive synergetic hierarchical framework that decouples strategic reasoning from tactical execution. Our architecture splits the workload between a “Strategic Brain” and a “Tactical Torso.” For the Brain, we utilize a synergy between DeepSeek-R1 (70B) and its 7B distilled counterpart to create a collaborative inference engine. By capitalizing on the inherent sparsity of tactical logic in air combat, we implemented a speculative decoding mechanism that achieves an effective boost in decision throughput while maintaining the deep logic of the full 70B model. For the Torso, we developed an enhanced MAPPO algorithm that processes relative pose graphs via graph attention. By integrating a KL-divergence constraint into the loss function, we essentially force agents with different payloads—like scouts and attackers—to evolve specialized tactical personalities within a shared latent space. Experimental results using the JSBSim high-fidelity 6-DOF engine demonstrate that the swarm does more than just improve its exchange ratio. Further t-SNE manifold analysis and Chain-of-Thought visualizations confirm that our architecture successfully aligns symbolic intent with raw physical control. Most notably, through our “decision-reflection-evolution” loop, the system proved it could diagnose its own failures, and iteratively refine its own tactical instructions.

## 1 Introduction

Modern aerial combat has pushed unmanned aerial vehicle (UAV) swarms past the point of being simple tools that just follow a script. We are seeing a shift where these clusters must act as autonomous tactical thinkers. In an arena defined by split-second changes and lopsided information, a basic set of flight commands just won't cut it anymore. The real challenge for multi-agent systems (MAS) and embodied AI researchers is figuring out how to let a swarm react to an enemy's move in milliseconds while simultaneously maintaining a high-level grip on the entire battlefield to plan for the long game (Li et al., 2025).

Currently, research is split into two distinct camps. On one side, we have end-to-end methods like Multi-Agent Reinforcement Learning (MARL). These are great at handling complex six-degree-of-freedom (6-DOF) maneuvers. For example, Zheng et al. (2024) used transfer learning to boost decision efficiency, and Wang et al. (2024) looked at evolutionary operators to handle swarms that change size on the fly. But these models often fall into a “short-sighted” trap. They lack a deep, conceptual understanding of air combat, such as Energy-Maneuverability theory. To make matters worse, newer generative models like MADiff (Zhu et al., 2024) create impressively smooth flight paths but suffer from high denoising latency. In a 50Hz flight control loop, that kind of delay is a dealbreaker.

On the other side, the academic world is testing Large Language Models (LLMs) as “strategic commanders” to give these agents more logical depth. Methods like Language Agent Tree Search (LATS; Luo et al., 2025) use Monte Carlo Tree Search (Swiechowski et al., 2023) to reach impressive levels of planning, and the Code-as-Policies approach (Liang et al., 2023) generates low-level code for flexible task matching. However, these don't translate well to the cockpit. A general LLM doesn't “feel” the airframe; it can't intuitively sense a stall boundary or a 6-DOF limit, which leads to disjointed movements. Plus, waiting several seconds for an LLM to think during a high-speed chase is a tactical disaster.

This massive gap between strategic depth and real-time execution is the main thing holding back UAV swarm intelligence in the real world (Javed et al., 2024). We started wondering: could we build an architecture that keeps the analytical power of a model like DeepSeek-R1 but retains the lightning-fast agility of reinforcement learning for low-level control? We saw how speculative decoding (Chen et al., 2023) sped up text generation in NLP and realized we could move that same idea over to the world of embodied games.

To bridge this divide, we developed a heterogeneous cognitive framework that pairs a “Strategic Brain” with a “Tactical Torso.” For the reasoning layer, we use a synergy between DeepSeek-R1 (70B) and its distilled 7B version. By applying speculative decoding, we've managed to cut down the wait time for semantic instructions enough to guide a live dogfight. For the execution layer, we built an enhanced MAPPO algorithm that decouples roles. By using a Graph Attention Network (GAT; Brody et al., 2022) to read the Relative Pose Graph (RPG) and a heterogeneity penalty based on KL-divergence, we've encouraged drones to develop complementary fighting styles in a shared latent space.

Our main contributions include:

1. We decoupled high-level strategic planning from low-level physical control across different time scales. This solves the problem of LLMs being “physically blind” and slow, while also fixing the tactical short-sightedness found in most RL-only setups.
2. Borrowing the idea of sampling alignment from recent studies (Chen et al., 2023; Zhang J. et al., 2024), we built a cloud-to-edge acceleration protocol. By letting the models work together, we hit a speedup ratio that finally allows LLMs to function in a high-stakes, high-speed environment.
3. We introduced a topology representation based on the RPG and an unconventional loss function using KL-divergence constraints. This forces different drones to pick up specialized roles—like scouts vs. attackers—within the same combat context, which directly improves the swarm's overall exchange ratio.
4. We utilized long-horizon reflection concepts from embodied AI (Luo et al., 2025; Tziafas and Kasaei, 2024) to let the LLM analyze failed flight paths. By figuring out the “why” behind a crash or a missed shot, the system automatically generates tactical corrections. We've shown that this helps drones teach themselves how to stay out of trouble in non-linear edge cases, like avoiding deep stalls.

The remainder of this paper is organized as follows: Section 2 reviews related work. Section 3 formulates the problem and environment. Section 4 details the proposed cognitive synergetic architecture. Section 5 presents the experimental results, and Section 6 concludes the paper.

## 2 Related works

### 2.1 Multi-agent reinforcement learning and swarm games

The landscape of UAV swarm games is currently shifting away from rigid, pre-programmed logic toward fully autonomous maneuvering and decision-making (Xu and Chen, 2022a,b). In high-pressure, changing environments, traditional reinforcement learning for a single agent often fails because it can't handle the dynamic nature of a group of drones. As a result, Multi-Agent Reinforcement Learning (MARL) has become the preferred method for researchers dealing with these challenging situations. To address the inherent non-stationarity and partial observability of these dynamic environments, the Centralized Training with Decentralized Execution (CTDE) paradigm has emerged as a foundational standard (Lowe et al., 2017). Under CTDE, a centralized critic leverages global state information during offline training to stabilize learning, while decentralized actors rely strictly on local observations for real-time execution. Building upon this paradigm, Multi-Agent Proximal Policy Optimization (MAPPO; Yu et al., 2022) has proven to be an exceptionally robust baseline. By extending the stable, clipped policy gradient updates of single-agent PPO into the multi-agent domain, MAPPO achieves superior sample efficiency and mitigates performance degradation in complex cooperative tasks. Given its proven stability, we selected MAPPO under the CTDE paradigm as the foundational reactive execution backbone for our architecture, subsequently enhancing it with graph attention and role-decoupling mechanisms to suit heterogeneous swarm combat.

Different schools of thought have emerged to solve the coordination problem. For instance, Zheng et al. (2024) used an Actor architecture with three subnetworks to facilitate knowledge transfer from simple duels to complex swarm combat. To prevent agents from getting stuck in “strategic cycles” and to handle the inevitable loss or addition of drones, Wang et al. (2024) and Hou et al. (2023) introduced evolutionary operators and attention mechanisms with death masking. While these tricks help, scaling up to truly large swarms still brings back the ghost of the “curse of dimensionality.” Mean field theory has stepped in to mitigate this (Hu et al., 2025; Wang et al., 2023) by treating individual interactions as a simpler game between one drone and the collective “average” of its neighbors, which effectively squashes the state space. Bio-inspired grouping mechanisms (Chi et al., 2023) and value decomposition networks (Gong et al., 2023) have also shown promise in getting drones to hunt as a pack. However, the robustness of MARL policies remains highly sensitive to the distribution of training opponents. To address this, Han et al. (2025a) proposed Prioritized Population Play with Diversified Partners (P3DPs), a landmark method that augments policy robustness by training agents against a spectrum of diversified opponent strategies using prioritized historical sampling. While P3DPs excels at building empirical robustness through massive data diversity, our framework offers a complementary, cognitive layer of defense: rather than relying solely on past exposure to diverse opponents, our Strategic Brain uses high-level reasoning to generalize counter-strategies against novel, unseen opponent behaviors in a zero-shot manner. Furthermore, to address the challenge of hybrid action spaces—where agents must simultaneously manage flight maneuvers and fire control—Han et al. (2025b) introduced a supervision-enhanced DRL framework. By utilizing a dual-head policy and supervised learning memory, this approach overcomes the limitations of sparse reward functions in BVR engagements. Our work shares this vision; while our Strategic Brain provides the macro-intent, the Tactical Torso's Bernoulli trigger mechanism can be further enhanced by incorporating such supervision-enhanced memory buffers to maximize the probability of kill in long-range missile exchanges.

### 2.2 Hierarchical decision-making and embodied intelligence

Hierarchical Reinforcement Learning (HRL) uses a “divide and conquer” approach to connect strategic planning with quick reactions. The usual route is to decouple target allocation from the actual maneuver execution (Chen et al., 2025; Xu et al., 2026; Yue et al., 2022). A notable example is the Leader-Follower strategy proposed by Pang et al. (2025), where wingmen estimate the leader's utility to tighten coordination within a 6-DOF space. In the broader world of Embodied AI, there is a growing push to bake physical laws directly into the learning process (Quero and Martinez-Carranza, 2024; Zhao and Liu, 2021). We'll see this in the use of Physics-Informed Neural Networks (PINNs) to estimate a UAV's non-linear dynamics (Bianchi et al., 2024) or indirect optimization methods for 6-DOF descent guidance (Nurre and Taheri, 2025).

Structuring the decision-making this way helps drop the dimensionality of the problem (Ren et al., 2021; Wang et al., 2021), yet it often results in a “black box” strategic layer. These rigid macro-action setups often fail when the combat situation takes a sharp, unexpected turn. High-level planning needs to be as adaptable as the low-level controls, yet current hierarchical models often lack the cognitive flexibility to rewrite their own playbook mid-flight.

### 2.3 Real-time cognitive engines driven by LLMS

The recent explosion in LLMs has given us models that can reason and associate concepts with a common-sense logic that was previously unheard of. Naturally, using an LLM as a robot's “brain” is now the frontier of Embodied AI (Kovalev and Panov, 2022; Qi and Jing, 2024). The “Code-as-Policies” approach (Liang et al., 2023) proved that LLMs can actually write the low-level control code themselves, and combining them with Monte Carlo Tree Search (MCTS) has produced agents with remarkable logical depth (Luo et al., 2025). But trying to put a large-parameter model in the cockpit of a fighter drone is an engineering nightmare because of the latency.

Some researchers are fine-tuning smaller LLMs for closed-loop control (Boyle et al., 2025), but even then, the delay is usually too high for millisecond-level aerial combat. This is where speculative decoding (Chen et al., 2023) changes the game. By letting a lightweight “draft” model sprint ahead and generate candidate tokens for a larger model to verify in parallel (Cheng et al., 2024; Zhang J. et al., 2024), we can finally think about real-time reasoning. Combined with TensorRT acceleration (Xu et al., 2024) and collaborative edge computing (Zhang M. et al., 2024), it is becoming possible to run these cognitive engines on embedded platforms like the NVIDIA Orin (Tian et al., 2025). By leveraging these acceleration techniques, we can finally give a drone cluster the “strategic pause” it needs without falling out of the sky.

### 2.4 Research gaps and motivation

If we look at the current literature, there is a persistent “contextual gap” between the two worlds, that is, MARL is getting better at generating fast actions, and LLMs are getting better at high-level reasoning, but they don't talk to each other very well. Pure LLM setups often hallucinate “un-physical” commands because they don't feel the airframe's limits, while pure RL setups lack the tactical “intuition” found in Energy-Maneuverability theory. We believe the answer lies in a unified architecture that uses speculative decoding to keep the “Brain” fast and intent embeddings to ground its thoughts in the “Torso's” physical reality. Our goal is to see a swarm that doesn't just react, but understands the physics of the hunt.

## 3 Problem formulation

In a high-stakes UAV swarm confrontation, the effectiveness of any decision-making system rests on two pillars: high-level tactical logic and the unforgiving physical constraints of the flight platform (Bjurling, 2025). We bridge these by coupling high-fidelity maneuver models with topology-aware graphs, creating a mathematical representation of air combat that remains grounded in real-world aerodynamics.

### 3.1 Heterogeneous swarm modeling and RPG representation

Our combat arena is a 3D space where red ($U_{R}$) and blue ($U_{B}$) teams of drones clash. To simulate the fast-paced nature of the battle, we model each drone $u_{i}\in U$ and its missiles using a complex 6-DOF system.

At any time step *t*, a drone's state *s*_*i, t*_ is a 12-dimensional vector:


$$\begin{array}{l} {\text{s}}_{i,t}={\left[{\text{p}}_{i,t}^{T},{\text{v}}_{i,t}^{T},\Phi_{i,t}^{T},\Omega_{i,t}^{T}\right]}^{T}\in {\mathbb{R}}^{12} \end{array}$$

Here, ${\text{p}}_{i,t}={\left[x_{i,t},y_{i,t},z_{i,t}\right]}^{T}$ anchors the drone in 3D space, while v_*i, t*_ tracks its linear velocity. The attitude Φ_*i, t*_=[φ_*i, t*_, θ_*i, t*_, ψ_*i, t*_]^*T*^ defines the roll, pitch, and yaw, and Ω_*i, t*_ measures the angular velocity.

We define the physical execution layer through a hybrid action vector a_*low, i, t*_=[δ_*a*_, δ_*e*_, δ_*r*_, δ_*t*_, *f*_*fire*_]^*T*^. We'll notice this includes both continuous aerodynamic controls—aileron (i.e., δ_*a*_), elevator (i.e., δ_*e*_), rudder (i.e., δ_*r*_), and throttle (i.e., δ_*t*_)—and a discrete trigger for weapons release (i.e., *f*_*fire*_; which is subsequently optimized by the execution layer discussed in Section 4.3).

To make sense of the swarm's shifting topology, we use a RPG. In this graph, nodes *v*_*i*_=[c_*type*_, *E*_*fuel*_, *N*_*missile*_, *S*_*radar*_]^*T*^ store individual “identity” and status—role type (scout, attacker, or decoy via one-hot encoding), fuel, ammo, and radar state. The directed edges *e*_*ij, t*_ are where the actual game geometry lives, encoding the relationship between agent *i* and its target *j*:


$$\begin{array}{l} e_{ij,t}={\left[d_{ij,t},q_{ij,t},\lambda_{ij,t},\Delta z_{ij,t}\right]}^{T} \end{array}$$

In this edge vector, *d*_*ij, t*_ is the Euclidean distance, which dictates weapon feasibility. The aspect angle *q*_*ij, t*_ measures how accurately the nose is pointed at the enemy (0° being a dead-on lock). The antenna train angle λ_*ij, t*_ tells us if the target is trying to run or heading for a face-to-face clash. Finally, Δ*z*_*ij, t*_=*z*_*j, t*_–*z*_*i, t*_ tracks the altitude gap, which is our primary metric for potential energy advantage.

We also found that static snapshots aren't enough for high-speed intercepts, so we include temporal rates of change to catch transient features. The range rateḋ_*ij, t*_is calculated as:


$$\begin{array}{l} {ḋ}_{ij,t}=\frac{{\text{r}}_{ij,t}\cdot \left({\text{v}}_{j,t}-{\text{v}}_{i,t}\right)}{d_{ij,t}} \end{array}$$

When this value is deeply negative, we know to watch out for overshooting the target. Crucially, we define**r**_*ij, t*_ = **p**_*j, t*_−**p**_*i, t*_as the relative position vector (Line-of-Sight vector) from agent *i* to target *j*. Similarly, the aspect angle rate${\dot{q}}_{ij,t}$helps us judge how stable our lock is, using the following derivative:


$$\begin{array}{l} {\dot{q}}_{ij,t}=\frac{\left({\text{v}}_{i,t}\times r_{ij,t}\right)\cdot \left({\dot{v}}_{i,t}\times r_{ij,t}+v_{i,t}\times {ṙ}_{ij,t}\right)}{||v_{i,t}{||}_{2}^{2}\cdot ||r_{ij,t}{||}_{2}^{2}\cdot \sin\left(q_{ij,t}\right)} \end{array}$$

#### 3.1.1 Partially observable markov game (POMG) modeling

Air combat is messy, and no agent ever has the full picture. Because of this “fog of war,” we formalize the confrontation as a POMG, defined by the tuple $<N,S,A,P,\vec{R},O,Z,\gamma >$.

Within this framework,represents all active drones on the field. The global state ${\text{s}}_{t}=\left\{{\text{s}}_{1,t},...,{\text{s}}_{|N|,t}\right\}$ contains every absolute position and hidden variable, but agents can't see this directly. Instead, they rely on a local observation space O—essentially a partial RPG subgraph limited by their sensor range. The joint action space $A\;=\;\phi A_{i}$ maps high-level intent down to those 6-DOF controls we mentioned earlier. The transition function P:S×A→Δ(S) determines how the world evolves, filtered through the laws of physics. We use Z as our observation function to model the probability of perceiving the environment given current signal-to-noise ratios, while the discount factor γε(0,1) helps us strike a balance between a quick tactical win and a long-term strategic advantage.

#### 3.1.2 Multi-objective reward formulation

Combat is essentially a balancing act between survival and lethality. To handle this, we define an individual reward as a three-dimensional vector ${\vec{R}}_{i,t}={\left[R_{surv},R_{kill},R_{ammo}\right]}^{T}$.

(1) Survival Reward (*R*_*surv*_): We penalize drones for being “painted” by enemy radar and for pushing the airframe too hard:
  $$\begin{array}{l} R_{surv}=-\exp\left(-\frac{d_{ij,t}^{2}}{\sigma_{s}}\right)\cdot I\left(q_{ji,t}<\alpha_{crit}\right)-\eta {||\Omega_{i,t}||}_{2} \end{array}$$ (1)
  Here, σ_*s*_ scales the threat based on distance, α_*crit*_ marks the critical threat angle where an enemy missile could lock on, and η is a penalty for high angular velocity—keeping the drone within a stable flight envelope and avoiding structural stress.
(2) Kill Reward (*R*_*kill*_): This is all about the “six o'clock” position. We use the RPG to guide the UAV into the ideal firing zone:
  $$R_{kill}=\{\begin{array}{l} \cos(q_{ij,t})\cdot \exp(-\left|d_{ij,t}-d_{opt}\right|),\;if\;q_{ij,t}\leq q_{\max}\\ -1,\;otherwise \end{array}$$ (2)
  The variable *d*_*opt*_ represents the optimal engagement range distance for a missile launch, and *q*_*max*_ is the limit of our radar's gimbal.
(3) Ammo Efficiency (*R*_*ammo*_):
To deter indiscriminate firing, we penalize low-probability weapon releases:
  $$\begin{array}{l} R_{ammo}=-f_{fire,i,t}\cdot \left(1-P_{k}\left(d_{ij,t},q_{ij,t}\right)\right) \end{array}$$ (3)
  In which, *f*_*fire, i, t*_ is our trigger bit, and *P*_*k*_ is the predicted kill probability at that specific moment.

## 4 Methodology

### 4.1 Cognitive synergetic hierarchical architecture

**Figure 1:**
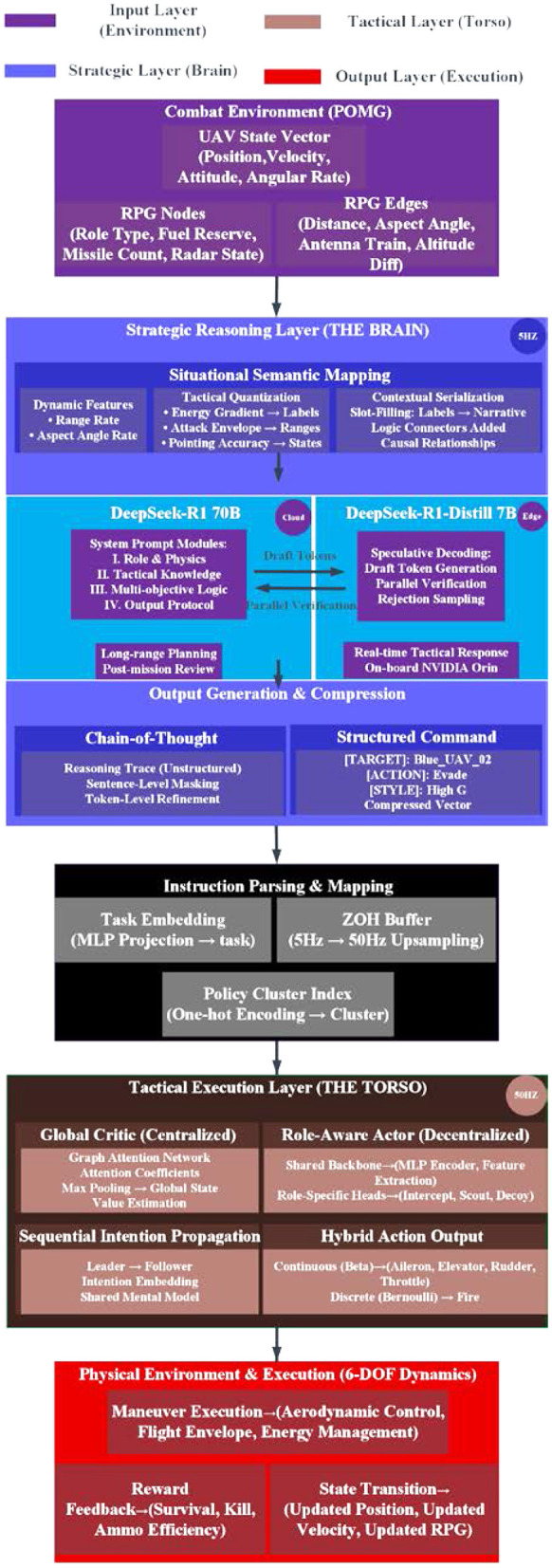
Overall architecture of the cognitive synergetic hierarchical framework for UAV swarm combat.

To navigate the inherent tension between high-level tactical planning and high-frequency physical response, we propose a modular cognitive framework. The overall data flow and functional decomposition of this system are illustrated in Figure 1. As we can see, the architecture is stratified into four distinct yet interconnected layers: the environment input layer (purple), the strategic reasoning layer (blue), the instruction parsing module (black), and the tactical execution layer (green). One might notice that this design effectively creates a dual-frequency control loop, where strategic intents generated at 5Hz are progressively grounded into 50 Hz aerodynamic commands through a Zero-Order Hold (ZOH) buffer and hybrid action heads. Traditional end-to-end reinforcement learning often falls into a “short-sighted” trap when dealing with long-duration tactical planning. Conversely, pure LLM setups struggle with the computational weight and latency required for millisecond-level aerodynamic compensation. To bridge this gap, we've developed a “Brain-Torso” hierarchical decision-making system. By decoupling high-level strategic intent from low-level precision control, we mimic the cognitive patterns of a human pilot while managing the computational load of heterogeneous tasks.

#### 4.1.1 “Brain-Torso” design and model selection

The core logic here is a clean split between “cold cognition” (strategic planning) and “hot cognition” (reactive control). This cognitive offloading is our way of sidestepping the curse of dimensionality inherent in high-dimensional state spaces.

#### 4.1.2 Strategic reasoning layer (the brain): distributed cognitive hub

For the high-level command center, we've tapped into DeepSeek-R1 (70B) and its distilled onboard counterpart. DeepSeek-R1′s intensive reinforcement learning background gives it a native Chain-of-Thought (CoT) capability. When it looks at the RPG from Section 3.1, it doesn't just see numbers; it reasons through complex, non-linear gambits like “luring the enemy deep” or “kinetic energy compensation.” To keep things running in real-time, we use a “Chief Designer and Assistant” setup: the full 70B model handles long-range planning and post-game review at the base station, while the DeepSeek-R1-Distill-Qwen-7B runs locally on the UAV's edge platform (i.e., NVIDIA Orin). This ensures the swarm maintains strategic depth without sacrificing second-by-second tactical responsiveness.

#### 4.1.3 Tactical execution layer (the Torso): reactive control via enhanced MAPPO

This layer acts as the reactive controller, built on an enhanced Multi-Agent Proximal Policy Optimization (MAPPO) algorithm. We've adopted the Centralized Training, Decentralized Execution (CTDE) framework and integrated role-based parameter decoupling. Once the “Torso” receives compressed tactical primitives from the “Brain,” it uses a policy network to find the physically optimal path within the 6-DOF constraints defined in Section 3. By confining RL exploration to the tactical subspace mapped out by the strategic layer, we've effectively solved the sparse reward problem typical in aerial combat.

### 4.2 Situational semantic mapping

The situational transformer, *f*_*text*_(.), is responsible for turning raw physical vectors s_*i, t*_ and RPG features *e*_*ij, t*_ into symbolic descriptions that actually mean something tactically. We build this through a progression of feature differentiation, expert-led quantization, and finally, semantic synthesis.

#### 4.2.1 Dynamic feature encoding and derivatives

We explicitly pull in temporal rates of change to identify high-level dynamic intents. For instance, we look at the range rate ḋ_*ij, t*_ to judge if we're losing our positional advantage or heading for an overshoot. We also track the aspect angle rate${\dot{q}}_{ij,t}$to evaluate how stable our nose-to-target tracking really is.

#### 4.2.2 Tactical quantization operator M and semantic space

We designed a quantization operator, M, to map continuous values into a discrete set of tactical labels L. These thresholds are derived from representative performance envelopes of Medium-Altitude Long-Endurance (MALE) UAVs (Fahlstrom et al., 2022) and the foundational principles of Energy-Maneuverability (E-M) theory (Boyd, 2018), which dictates how energy state transitions influence combat potential. Following standard tactical air-to-air doctrine (National Research Council, 2005; Shaw, 1985), the quantities are defined to represent critical operational boundaries:

Potential Energy Gradient (Δ*z*_*ij, t*_→*L*_Δ*z*_): Δz ≥ 1000 m indicates an energy advantage; between (−1000,1000)*m* is a neutral state; and ≤ -1000*m* is a serious energy deficit.

Attack Envelope (*d*_*ij, t*_→*L*_*dist*_): Based on standard missile weapon engagement zones (WEZ) (Shaw, 1985), (0,5)*km* is Within Visual Range (WVR) combat; (5,30)*km* is Medium Visual Range (MVR) engagement; and (30,80)*km* is the edge of radar tracking.

Pointing Accuracy (*q*_*ij, t*_→*L*_*aspect*_): Representing the field-of-view limits of typical radar and missile seekers (National Research Council, 2005), (0,30°) is an offensive lock; (30°,90°) is a maneuvering approach; and ≥90° is a defensive posture.

Dynamic Trend (ḋ_*ij, t*_→*L*_*rate*_): < -50*m/s* is rapid closure; [−50,50]*m/s* is range maintenance; and >50*m/s* is rapid separation.

### 4.3 Structured prompt templates and command generation

To keep DeepSeek-R1′s reasoning within the bounds of physics, we developed a Dynamic Prompt Generation Engine. Instead of static text, this engine uses a real-time state machine to produce a composite prompt$P_{total}$ containing cognitive anchoring, situational narratives, and hard-constrained instructions.

#### 4.3.1 System prompt architecture

The system prompt $P_{sys}$ builds the agent's “tactical ego” using four locked modules:

Module I (Role and Physics): anchors the model as a “Cognitive Commander” bound by 6-DOF dynamics and E-M theory.

Module II (Tactical Knowledge): injects specific maneuvers like the “Notch Maneuver” to break locks or “High-YoYo” to trade speed for altitude.

Module III (Multi-objective Logic): forces the model to weigh Survival, Kill Chain, and Energy against each other.

Module IV (Output Protocol): strictly mandates a Reasoning Trace followed by a bracketed Command Block.

#### 4.3.2 Contextual serialization

LLMs aren't naturally great with raw numbers, so we use a slot-filling algorithm to turn discrete labels L into a causal narrative $P_{ctx}$. We might fill a slot like: “Self-Unit 01 is in Severe_Energy_Deficit state.” Then, we add logic connectors based on derivatives. If altitude is dropping while the enemy is closing in, the engine might insert: “WARNING: This indicates an imminent high-energy dive attack.” This forces the model to focus on causal relationships rather than isolated data points. To enhance the reproducibility of our framework, we explicitly provide the architectural template of the integrated prompt generated at each decision step, as shown in Box 1.

Box 1 Explicit Prompt Template Integrating Scenario Information.

**[SYSTEM ROLE]**

You are the Tactical Brain of Blue_UAV_01. Your goal is to maximize the exchange ratio while ensuring survival. You are bound by 6-DOF dynamics and E-M theory.

**[ENVIRONMENT STATE (Slot-Filled from RPG)]**

Self Status: {Energy_State: [Insert Quantized Δ*z* fromL], Speed_Trend: [Insert Range Rate fromL]}

Target (Enemy_01): Distance is {*d*_*ij*_} km. Aspect Angle is {*q*_*ij*_} degrees.

**[DYNAMIC WARNINGS]**

{IF Range_Rate < −50 AND Aspect_Angle < 30}:

“WARNING: Enemy is closing rapidly with a nose-on lock. Immediate evasion required.”

**[KNOWLEDGE RETRIEVAL]**

{IF Energy_Deficit}:

“Consider Horizontal S-turns to break lock without losing speed.”

**[INSTRUCTION]**

Analyze the causal relationships above. Output your reasoning trace exactly as [THOUGHTS]: < text>, followed immediately by the command block using the exact tags:

[ACTION]: < Evade/Intercept/Scout>

[STYLE]: < High G/Smooth/Maintain>

#### 4.3.3 Dual structure of T_*out*_

The strategic layer's output, *T*_*out*_, is formalized as an ordered tuple: $<S_{CoT},S_{CMD}>$

Reasoning trace ($S_{CoT}$): this is the unstructured thinking process. It's useful for us to look at, but its real job is providing the “why” for our reflection mechanism.

Structured command block ($S_{CMD}$): this is the hard-coded interface at the end of the text. It is strictly constrained to a bracketed Key-Value protocol (e.g., [KEY]: Value) to ensure deterministic parsing by the execution layer. We'll find things like [TARGET] (which enemy to focus on), [ACTION] (a categorical variable such as Evade, Intercept, or Scout used for role-gating), and [STYLE] (a state parameter, such as High G, dictating how aggressive the execution should be).

### 4.4 Instruction parsing and mapping

The parsing layer, Parse(.), translates the text *T*_*out*_into a machine-readable input a_*high*_=[i_*cluster*_, e_*task*_], which eventually drives the physical control signals.

#### 4.4.1 Policy cluster indexing and safety validation

We use regular expressions to grab the POLICY_CLUSTER and map it to a one-hot encoding i_*cluster*_. To mitigate the inherent hallucination risks of general-purpose LLMs, this parsing layer acts as a hard-coded safety filter. If the LLM generates an out-of-bounds action or an invalid format, the filter rejects the command, and the ZOH buffer sustains the previous valid intent. When valid, this index acts as a gate, activating the specific “Intercept” or “Scout” behavior branch in the underlying MAPPO network.

#### 4.4.2 Task-conditioned embedding

To pass along subtle intent (like the RISK_BIAS), we extract the hidden state h_*llm*_ from the edge model. This vector is projected into a latent space *via* a Multi-Layer Perceptron (MLP):


$$\begin{array}{l} {\text{e}}_{task}=\sigma \left(W_{proj}\cdot {\text{h}}_{llm}+b_{proj}\right) \end{array}$$ (4)

This embedding e_*task*_ captures the nuanced execution characteristics of the maneuver, such as its aggressive posture and risk tolerance.

#### 4.4.3 Execution and physical action generation

These high-level components are fed into the MAPPO networks where the Actor network fuses local observations o_*i, t*_ with the task embedding:


$$\begin{array}{l} {\text{a}}_{low,i,t}\sim \pi_{i}\left(\cdot |{\text{o}}_{i,t}⊕{\text{e}}_{task},{\text{i}}_{cluster}\right) \end{array}$$ (5)

The result, a_*low, i, t*_, is the 5-dimensional hybrid vector [δ_*a*_, δ_*e*_, δ_*r*_, δ_*t*_, *f*_*fire*_]^*T*^ defined in Section 3.1. To handle this mixed action space, we implement a hybrid policy head architecture that branches from the Actor's backbone to parameterize different probability distributions simultaneously. Specifically, for the four aerodynamic control surfaces [δ_*a*_, δ_*e*_, δ_*r*_, δ_*t*_], the network outputs the α and β parameters of independent Beta distributions. Unlike standard Gaussian distributions which require heuristic clipping, Beta distributions are naturally bounded within [0,1]. This ensures that generated commands never exceed the physical deflection limits of the airframe, preventing gradient bias during backpropagation. For the weapon trigger bit *f*_*fire*_, the network outputs a single probability parameter for a Bernoulli distribution. This provides a mathematically rigorous way to model the binary nature of fire control (0 for hold, 1 for release) while maintaining differentiability through the policy gradient (Yu et al., 2022).

#### 4.4.4 Asynchronous alignment

There's a significant frequency mismatch here: the LLM thinks at roughly 5 Hz, but the flight controller needs 50 Hz. We handle this with a Zero-Order Hold (ZOH) strategy. This specific architectural choice acts as a low-pass filter for intent, preventing the high-frequency control chattering that would inevitably occur if aerodynamic surfaces were continuously perturbed by the intermediate, token-by-token generation process of the LLM. The Actor network keeps using the most recent e_*task*_ and i_*cluster*_ for its 50 Hz inferences until the “Brain” provides a fresh strategic update. This keeps the flight stable even while the “Brain” is still crunching the next tactical move.

### 4.5 Strategic reasoning layer: a real-time cognitive engine via large-small model synergy

Aerial combat moves at a breakneck pace, often leaving a decision window of only a few hundred milliseconds. While the DeepSeek-R1 (70B) model introduced in Section IV.A offers formidable reasoning power, the time required to generate a full Chain-of-Thought (CoT) is simply too long for live engagement. We bridge this gap between “logical depth” and “inference speed” by introducing a real-time cognitive engine built on logic compression and speculative decoding.

#### 4.5.1 Two-stage logic compression

To make these models viable for resource-constrained onboard systems, we condense verbose text into a high-density sequence of tactical primitives through a two-stage compression algorithm.

1. Sentence-level semantic masking

The first step is to isolate “logic anchors”—sentences that actually drive the tactical conclusion. Given an original CoT sequence$C=\left\{s_{1},s_{2},...,s_{N}\right\}$, we use an adversarial perturbation test to score each sentence *s*_*k*_ based on its importance, Ω(*s*_*k*_):


$$\begin{array}{l} \Omega \left(s_{k}\right)=D_{KL}\left(\pi \left({\text{a}}_{high}|C\right)||\pi \left({\text{a}}_{high}|C\backslash \left\{s_{k}\right\}\right)\right) \end{array}$$ (6)

Here, $D_{KL}$ is the Kullback-Leibler divergence, and π(a_*high*_ |.) is the action distribution output by the parsing layer. Basically, if removing a sentence *s*_*k*_ causes a massive shift in the resulting action distribution (i.e., Ω(*s*_*k*_)>δ_*th*_), that sentence contains core game logic—like “aborting pursuit due to low energy”—and must be kept. Everything else is treated as redundant and pruned.

2. Token-level refinement and mapping templates

Once we have our key sentences, we use a slot-filling template to turn natural language into a compact structural vector c_*compressed*_. We've defined three independent semantic dimensions: <O,D,U>

O (Operation): tactical intent, selected from {‘Engage', ‘Evade', ‘Flank', ‘Maintain', ‘Return'}.

D (Directionality): spatial orientation, selected from {‘Clock_2', 'Clock6', ‘Left/Right_Turn', ‘Climb/Dive', ‘Notch'}.

U (Urgency): execution priority, selected from {‘High', ‘Medium', ‘Low'}, which maps to energy consumption preferences.

For instance, if the model says, “*Enemy missile detected at 4 o‘clock. Break right immediately and dive*,” our compressor Ψ_*compress*_ fills the slots: *Break right* becomes D= Right|Dive, *Immediately* becomes U= High, and *missile* triggers O=EvadeO=Evade. This outputs a vector c_*compressed*_=[OP:Evade,DIR:Right|Dive, URGENCY:High]. We'll find this reduces token usage by about 60%, easing the load on the parser while keeping the causal chain intact for the reflection mechanism.

#### 4.5.2 Speculative Inference Synergy

To get 70B-level intelligence at 7B-level speeds on the edge, we've built a “cloud-to-edge” inference pipeline based on speculative decoding.

1. Heterogeneous deployment

Draft Model (*M*_*draft*_): Running on the UAV (i.e., an NVIDIA Orin), we use a DeepSeek-R1-Distill-Qwen-7B that has undergone full instruction tuning. It's tuned to understand the complex $P_{total}$ prompt structure from Section 4.1.3, ensuring it generates candidate tokens at a high rate.

Target Model (*M*_*target*_): The full DeepSeek-R1 (70B) sits at the ground station. Instead of generating text word-by-word, it verifies the draft sequences in parallel.

2. Speculative sampling and verification

At each step *t*, the synergy follows a specific protocol. First, *M*_*draft*_ “guesses” a sequence of length *L*:


$$\begin{array}{l} {ŷ}_{i}\sim P_{draft}\left(y_{i}|{ŷ}_{<i},P_{total}\right) \end{array}$$ (7)

Then, *M*_*draft*_ performs a single forward pass to verify these tokens. We use a rejection sampling scheme to decide if we accept each tokenŷ_<*i*_:


$$\begin{array}{l} r\sim U\left[0,1\right],\;Accept\;if\;r<\min\left(1,\frac{P_{target}\left({ŷ}_{i}|{ŷ}_{<i},P_{total}\right)}{P_{draft}\left({ŷ}_{i}|{ŷ}_{<i},P_{total}\right)}\right) \end{array}$$ (8)

As long as the small model's logic aligns with the large model, we keep the tokens. If they diverge, we fall back to the 70B model to correct the sequence. It is crucial to emphasize that this entire process occurs internally within the cognitive engine. The “draft” decisions generated by the 7B model are strictly speculative and are never directly transmitted to the UAV. Only the final, fully verified sequence—guaranteed to match the 70B model's distribution—is parsed and sent to the Tactical Torso. This strict “verify-before-execute” mechanism ensures that potentially flawed logic from the smaller model cannot cause catastrophic flight consequences.

3. Theoretical speedup

This works because aerial combat logic has “sparse complexity.” Most of the time, the moves are predictable and the 7B model handles them fine; the 70B model only needs to step in during high-stakes tactical shifts. If we let α be the average acceptance rate, the expected speedup ratio η is:


$$\begin{array}{l} \eta =\frac{\tau_{target}}{\tau_{draft}+\left(1-\alpha \right)\tau_{target}/L} \end{array}$$ (9)

Where *L* is the speculative step length and τ represents the average latency for a single token from each model.

### 4.6 Tactical execution layer: swarm reinforcement learning and coordination

If the strategic layer is the “Brain,” the tactical execution layer is the “Muscles.” We use a Role-Decoupled MAPPO framework paired with a sequential intention propagation mechanism to stop heterogeneous swarms from drifting into homogeneous, ineffective behaviors.

#### 4.6.1 Role-decoupled heterogeneous policy network

Standard MARL often assumes agents are identical, but in a swarm comprising scouts and attackers, this leads to a suboptimal, generalized strategy. We employ the CTDE paradigm specifically to alleviate the non-stationarity of the environment while respecting the communication limits of a real battlefield. During the training phase, CTDE permits a centralized Critic to access the global state *s*_*t*_—including all agents' hidden states and the complete RPG topology—to accurately estimate the value function. During live execution, the decentralized Actors rely exclusively on their localized observations o_*i, t*_ and the Brain's instructions. This design choice guarantees operational viability even under severe communication latency or jamming, a necessity that is empirically validated in our supplementary ablation study (see Section 5.4.4).

1. Global critic with graph attention

To handle the non-stationarity of the environment, our Critic network$V_{\phi }\left({\text{s}}_{t},G_{t}\right)$uses a Graph Attention Network (GAT). This allows the model to digest the RPG from Section 3.1, which is essential because drones might get shot down or damaged, changing the graph size dynamically.

For any agent *i*, we calculate attention coefficients α_*ij*_ for its neighbors:


$$\begin{array}{l} \alpha_{ij}=\text{Softma}{\text{x}}_{j}\left(\text{LeakReLU}\left({\text{w}}^{T}\left[\text{W}{\text{h}}_{i}||\text{W}{\text{h}}_{j}||e_{ij,t}\right]\right)\right) \end{array}$$ (10)

In which, W is our weight matrix and *e*_*ij, t*_ represents the edge features. After pooling these features via Max-Pooling to create a global state vector g_*t*_, the Critic outputs a value estimate *V*_φ_ = MLP(g_*t*_).

2. Role-aware actor

The Actor network π_θ_(a_*low, i, t*_ |.) uses a “shared backbone, independent heads” design.

Shared Backbone: All agents use the same MLP encoder to pull raw physical features from their observations o_*i, t*_.

Role-Gating: Based on the POLICY_CLUSTER index i_*cluster*_ from the Brain, we activate specific policy heads. If the Brain says ‘Intercept', the drone uses a head trained for high-G maneuvers; if it says ‘Scout', it switches to a head optimized for smooth, wide-area radar coverage.

Hybrid Outputs: We use Beta distributions for the 4-DOF control surfaces and a Bernoulli distribution for the weapon trigger (*f*_*fire*_), optimizing continuous and discrete actions together. The strategic choice of Beta distributions over standard Gaussians is critical here; Beta distributions provide strictly bounded continuous outputs, which perfectly map to the absolute physical deflection limits of aerodynamic control surfaces [e.g., (−1,1)], effectively preventing the generation of un-physical out-of-bound control signals without requiring artificial gradient clipping. In extending this architecture to BVR engagements, we align our dual-head structure with the supervision-enhanced paradigm proposed by Han et al. (2025a). Specifically, the maneuvering head utilizes Beta distributions for continuous control, while the fire control head can be optimized by integrating binary cross-entropy loss against historical missile hit outcomes. This paradigm allows the “Torso” to predict kill probabilities concurrently with maneuver generation, enabling the swarm to transition seamlessly from WVR dogfights to BVR missile exchanges.

#### 4.6.2 Sequential intention propagation (SIP) and shared mental models

The hardest part of swarm combat is making sure everyone is on the same page. We use SIP to build a Shared Mental Model.

Instead of everyone acting at once, we use the TARGET_ID and roles to set a cascaded decision sequence

$S_{seq}=\left(u_{\left(1\right)},u_{\left(2\right)},...,u_{\left(N\right)}\right)$ that is resolved entirely within a single 20 ms execution step (50 Hz). To clarify the temporality, this is an auto-regressive process where agents compute actions sequentially to prevent conflicting maneuvers. The intra-swarm communication latency for broadcasting these embeddings is at the sub-millisecond level, ensuring the entire chain is completed before physical actuation.

Topologically, the broadcasting follows a one-to-all cascaded graph: as shown in Equation (15), the (*k*+1)-th agent does not merely listen to the “Leader,” but integrates the weighted sum of intentions from all *k* preceding agents in the sequence. The Leader (*u*_(1)_) outputs its intention embedding m_(1), *t*_, which is broadcasted to all subsequent peers. Each following agent then updates the “Shared Mental Model” by adding its own intended vector to the pool. The (*k*+1)-th agent's policy is then conditioned on this cumulative latent representation:


$$\begin{array}{l} {\text{a}}_{low,k+1,t}\sim \pi_{k+1}\left(\cdot |{\text{o}}_{k+1,t},\underset{\text{Shared}\;\text{Mental}\;\text{Model}}{\underset{︸}{\overset{k}{\underset{j=1}{\sum }}\alpha_{j}{\text{m}}_{\left(j\right),t}}}\right) \end{array}$$ (11)

#### 4.6.3 Loss function and optimization

Our composite loss function *L*(θ) balances performance, exploration, and role-specific behavior:


$$\begin{array}{l} L(\theta )={\text{E}}_{t}[L_{CLIP}(\theta )-c_{1}L_{VF}(\phi )+c_{2}S(\pi )\\ \;-\lambda_{div}D_{KL}(\pi_{intercept}||\pi_{scout})] \end{array}$$ (12)

It's standard PPO at its core—*L*_*CLIP*_ keeps updates stable and *L*_*VF*_ trains the Critic. However, the crucial component is the regularization term:$D_{KL}$. We use it as a “repulsive” force to maximize the difference between roles. This prevents non-combat units, such as scouts, from deviating into aggressive combat roles, thereby ensuring the swarm maintains optimal specialization and operational efficiency.

#### 4.6.4 Decoupled hierarchical training procedure

To clarify the learning dynamics of our hierarchical architecture, we explicitly adopt a decoupled, two-phase training procedure. The Strategic Brain and the Tactical Torso are not trained end-to-end simultaneously.

Phase 1: strategic brain preparation (offline). The large target model, DeepSeek-R1 (70B), is deployed in its pre-trained state with its weights remaining completely frozen. To prepare the edge-deployed draft model (DeepSeek-R1-Distill-Qwen-7B), we perform offline Supervised Fine-Tuning (SFT) using a curated dataset of high-quality tactical reasoning trajectories previously generated by the 70B model. This offline tuning ensures the 7B model strictly adheres to our structured prompt template (Box 1) and JSON-like output formats. During the subsequent RL phase, the weights of both LLMs are strictly frozen.

Phase 2: tactical Torso RL training (online). The MAPPO Actor and Critic networks (the Torso) are trained from scratch through direct interaction with the JSBSim environment. During this phase, the frozen Strategic Brain acts purely as a conditional intent generator: it processes the RPG at 5 Hz and outputs the policy cluster index i_*cluster*_ and task embedding e_*task*_. The Torso treats these LLM outputs as high-level environmental conditions. Operating at 50 Hz, the Torso collects experience trajectories (o_*i, t*_, e_*task*_, a_*low, i, t*_, *r*_*i, t*_) guided by the multi-objective rewards defined in Section 3.3. The policy gradients are then calculated to update only the neural weights of the MAPPO Torso (θ and φ) using the composite loss function *L*(θ) defined in Equation 16. This approach allows the Torso to master 6-DOF physical execution while being steadily guided by the invariant, zero-shot logical priors of the Brain.

### 4.7 Theoretical analysis

We need to be sure that this framework isn't just a collection of clever heuristics but is actually grounded in mathematical reality. To that end, we've analyzed the feasibility and convergence of the system from two angles: the hard limits of real-time computation and the stability of the policy optimization itself.

#### 4.7.1 Inference latency and communication complexity

In a “Brain-Torso” setup, the biggest risk is that the “Brain” takes too long to think, leaving the “Torso” to fly blind. System stability effectively boils down to whether our delays violate the Nyquist frequency constraints of the control loop.

Theorem 1 (Sufficient Conditions for Real-time Stability). Let *F*_*ctrl*_ represent the minimum update frequency required by the low-level flight controller, and let *T*_*dyn*_ be the shortest time window in which the tactical situation undergoes a qualitative shift (for instance, a target breaking a lock). The closed-loop system remains stable if:

Execution Layer Constraint:$\tau_{RL}<\frac{1}{F_{ctrl}}$

Strategic Layer Constraint:$\frac{\tau_{LLM}}{\eta }+\tau_{comm}<T_{dyn}$

where τ_*RL*_ and τ_*LLM*_ are the single-step inference times for the tactical and strategic layers, respectively; τ_*comm*_ is the communication latency; and η is the speedup ratio from speculative decoding defined in Section 6.2.2.

Proof:

We can look at this through the lens of a dual-frequency loop. On the execution side—the high-frequency loop—our MAPPO policy network is a standard MLP with *L* layers, an input dimension *d*_*in*_, and a hidden width *d*_*h*_. If you run the numbers, the floating-point operations (FLOPs) are roughly:


$$\begin{array}{l} C_{RL}=2\cdot L\cdot d_{h}^{2}+2\cdot d_{in}\cdot d_{h} \end{array}$$ (13)

For a typical lightweight setup (*L* = 3, *d*_*h*_= 256) deployed on an NVIDIA Orin with a peak performance of *P*_*peak*_, the inference time τ_*RL*_ is around 0.5*ms*. Since a 50Hz flight controller needs a 20ms cycle, 0.5ms < < 20ms means the execution layer handles its workload with plenty of room to spare.

The low-frequency loop is where it gets tricky. The strategic layer is a Transformer, which means we're dealing with *O* (*N*^2^) self-attention complexity. A raw 70B model might take over a second to generate a full tactical CoT (τ_*LLM*_). However, our speculative decoding slashes this to τ′=τ_*LLM*_/η. In the physics of a dogfight, the situational coherence time *T*_*dyn*_ is essentially the ratio of relative distance *D* to relative velocity *V*_*rel*_. At close range, say 500m at 300m/s, *T*_*dyn*_is about 1.6s. If our speedup η ≥ 2.0 and we keep communication lag τ_*comm*_ under 100ms, the total strategic delay stays around 1.1s. This is comfortably under 1.6s, ensuring the “Brain” sends its intent while the tactical context is still valid.

#### 4.7.2 Monotonic improvement in heterogeneous policy optimization

One might worry that adding a specific penalty for role similarity—our$-\lambda_{div}D_{KL}$term—might prevent the agents from actually learning how to win. We can prove that the algorithm still guarantees monotonic policy improvement under certain bounds.

Theorem 2 (Lower Bound for Heterogeneous PPO Improvement). Let *J*(π) be the expected cumulative reward for a policy π. For a heterogeneous MAPPO update step, the improvement of a new policy π_*new*_ over an old one π_*old*_ satisfies:


$$\begin{array}{l} J\left(\pi_{new}\right)-J\left(\pi_{old}\right)\geq \underset{Standard\;PPO\;Bound}{\underset{︸}{\text{E}\left[\frac{\pi_{new}}{\pi_{old}}A^{\pi_{old}}\right]-C\cdot \varepsilon_{KL}}}-\lambda_{div}\cdot \Delta_{H} \end{array}$$ (14)

Here, $A^{\pi_{old}}$is the advantage function, *C* is a constant related to the value function, ε_*KL*_is the upper bound on the KL divergence between new and old policies, and Δ_*H*_ is the maximum shift in the heterogeneity penalty. Monotonic convergence is guaranteed as long as the regularization coefficient $\lambda_{div}\leq \frac{\delta_{adv}}{\Delta_{H}}$, where δ_*adv*_ is the minimum advantage gain from a strategic improvement.

Proof:

Starting with the original PPO bound established by Schulman et al. (2017), we know that maximizing the surrogate objective is equivalent to optimizing a lower bound on the true reward:


$$\begin{array}{l} J\left(\pi_{new}\right)\geq J\left(\pi_{old}\right)+\underset{\text{s}}{\sum }\rho_{\pi_{old}}\left(\text{s}\right)\underset{\text{a}}{\sum }\pi_{new}\rho_{\pi_{old}}\left(\text{a}|\text{s}\right)A^{\pi_{old}}\left(\text{s},\text{a}\right)\\ -C\cdot \underset{\text{s}}{\max}D_{KL}\left(\pi_{old}||\pi_{new}\right) \end{array}$$ (15)

In this expression,ρ_π_*old*__represents the state visitation distribution.

Our objective adds the heterogeneity term$L_{div}=-\lambda_{div}D_{KL}(\pi_{intercept}||\pi_{scout})]\$.Ifwedefin\$\Delta_{H}=\left|L_{div}(\pi_{new})-L_{div}(\pi_{old})\right|$. If we definΔ_*H*_ = |*L*_*div*_(π_*new*_)−*L*_*div*_(π_*old*_)|as the change in this penalty during an update, we can see that this term acts as a bounded perturbation. We can rewrite the total expected reward as:


$$\begin{array}{ll} J_{total}\left(\pi_{new}\right)\geq J_{PPO\text{\_}Bound}-\lambda_{div}\cdot \underset{\theta }{\max}||\nabla_{\theta }D_{KL}\left(\pi_{\mathrm{int}}||\pi_{scout}\right)||\\ \;\cdot & \alpha \end{array}$$ (16)

where α is the learning rate. For the total reward *J*_*total*_to keep increasing, the primary push from the policy gradient must be stronger than the pull of the regularization:$E\left[A^{\pi_{old}}\right]>\lambda_{div}\cdot \Delta_{H}$. As long as we keep λ_*div*_ small enough—or decay it over time—the heterogeneity constraint won't derail the gradient direction. This mathematical safety net is why we use a dynamic λ_*div*_ decay in our actual experiments (parameter configurations are specified in Section 5.1.5), allowing the swarm to settle into distinct, optimal roles without losing sight of the mission goal.

## 5 Experiment

### 5.1 Experimental setup

#### 5.1.1 Simulation platform

We moved away from simplistic point-mass models (Imado et al., 2011; Kinoshita and Imado, 2006) and instead built our environment on the JSBSim flight dynamics engine to capture the true nuances of aerial combat. By loading non-linear 6-DOF aerodynamic data for the F-16, we can accurately simulate complex physical traits like aerodynamic coupling at high angles of attack, energy bleed during maneuvers, and throttle lag. Building on this core, we developed a Partially Observable Markov Game (POMG) interface that supports heterogeneous multi-agent setups. We also integrated line-of-sight radar detection models (PoD) and electronic jamming to replicate the friction of a real-world battlefield.

To test the “Brain-Torso” architecture on hardware people can actually buy, we used a split deployment: a high-performance workstation for the “Brain” and an embedded edge device for the “Torso.” For the Strategic Reasoning Layer (Brain), we handled the massive memory needs of DeepSeek-R1 by using a workstation with dual NVIDIA GeForce RTX 3090 (24GB × 2) GPUs. Running the DeepSeek-R1-Distill-Llama-70B model required us to use 4-bit AWQ quantization. On the other end, the Tactical Execution Layer (Torso) lived on an NVIDIA Jetson AGX Orin (64GB). This edge platform ran a quantized DeepSeek-R1-Distill-Qwen-7B alongside our MAPPO Actor network. We used the TensorRT engine to hit a 50Hz refresh rate, turning macro-level strategy into immediate control signals. To make things even more realistic, we modeled the communication between the cloud and the edge as a Gaussian distribution (mean 50ms, std 10ms), which lets us see how the swarm handles network jitters and lag.

The physical constraints, sensor specs, and game rules are all detailed in Table 1 for those interested in reproducing these results.

**Table 1:** Key physical parameters and rules of the simulation environment.

Category	Parameter	Value/Setting
Dynamics	Flight platform	F-16 Falcon (Non-linear 6-DOF)
	G-Load limits	+9.0G/-3.0G
	Simulation frequency	50 Hz (Physics), 10 Hz (Decision)
	Fuel load	3200 lbs (Standard internal)
Sensors	Radar range	80 km (Lock), 150 km (Search)
	Radar gimbal limit	±60°
	Visual range	15 km
Rules	Missile range (*R_*max*_*)	30 km (AIM-120 Simulation)
	No-escape zone (*R_*ne*_*)	12 km
	Kill Condition	Distance < 50 m and Aspect < 30°

#### 5.1.2 Confrontation scenarios

We tested the adaptability of our framework across three scenarios of increasing difficulty, as shown in Table 2. In all cases, the “Blue” side uses a competitive Behavior Tree expert system. Its logic follows standard AFTTP 3-3 tactical manuals (Air Force Space Command, 2016), making it a formidable opponent that moves like a seasoned human pilot. Furthermore, to address the potential limitation of evaluating against a single fixed strategy, we established an “Adversarial Pool” for cross-play evaluation. In addition to the AFTTP expert system, this pool includes an RL-based opponent (a well-trained MAPPO swarm) and an Evolutionary-based opponent (Wang et al., 2024), covering diverse and unpredictable tactical behaviors.

**Table 2:** Summary of configuration details for air combat scenarios.

Scenario	Scale	Heterogeneous roles	Complexity	Key metric
A. Dogfight	1 vs. 1	Homogeneous	Physical Limits	*η_*e*_, Jerk*
B. Hetero-Coop	2 vs. 2	Scout + Attacker	Role Symmetry	*ER*,$D_{KL}$
C. Swarm-War	6 vs. 6	Mixed Roles	Jamming + Lag	*WR, η*

Scenario A: 1v1 Within-Visual-Range (WVR) Dogfight focuses on the marriage of 6-DOF boundary control and energy-maneuverability theory. Drones start head-on at 10km. The goal is to perform high-G maneuvers like the High-YoYo to get into a firing position without stalling. This acts as a stress test for our physical control stability.

Scenario B: 2v2 Heterogeneous Cooperation checks if our role-gating actually works. The Red side has one “Attacker” (with missiles) and one “Scout” (high mobility, radar, but no weapons). The scout has to act as bait and share data, while the attacker takes the long-range shot. If they both try to do the same thing—like the scout trying to dogfight—the mission usually fails.

Scenario C: Scale Swarm Combat is the final test for long-range planning. This 6v6 fight includes attackers, scouts, and jammers. We used “observation masking” where jammers kill radar PoD within 20km and applied our 50ms Gaussian network delay. It forces the “Brain” to act like a grandmaster, coordinating units while dealing with a degraded network.

#### 5.1.3 Baseline algorithms

We compared our framework against six state-of-the-art methods across the Multi-Agent System (MAS) and Embodied AI spectrum, and specialized UAV air combat domains:

1. HAPPO (Kuba et al., 2021): A classic for credit assignment in heterogeneous groups. We use it to see if our role-gating adds any real value.
2. MAT (Wen et al., 2022): This treats MARL as a sequence modeling problem. It's powerful for dependencies but can get sluggish as swarms grow due to *O*(*N*^2^) complexity.
3. MADiff (Zhu et al., 2024): Uses diffusion models for smooth trajectories. It looks great on paper but the 20-50 denoising steps make it too slow for the 50Hz loops we need.
4. LATS (Zhou et al., 2023): Combines LLMs with Monte Carlo Tree Search. It's the gold standard for logic but takes seconds to make one decision. We use it to benchmark our Strategic Layer.
5. CaP (Liang et al., 2023): The popular industry choice where LLMs write Python code to drive controllers. We want to show that our neural approach is more robust when things get “messy” (like dodging missiles) compared to rigid generated code.
6. Zheng et al. (2024): A specialized MARL algorithm designed specifically for UAV swarm air combat maneuvers using transfer learning. We include this to benchmark our framework against domain-specific air combat RL solutions.

#### 5.1.4 Metrics

We use three types of metrics to catch issues like physical instability or computation lag.

1. Combat effectiveness: We evaluate combat effectiveness primarily through the Exchange Ratio (*ER*), a fundamental air combat metric defined as the ratio of enemy units destroyed to friendly units lost:

$$\begin{array}{l} ER=\frac{{\bar{N}}_{kill}+1}{{\bar{N}}_{loss}+1} \end{array}$$ (17)

Where ${\bar{N}}_{kill}$ and ${\bar{N}}_{loss}$ are average kills and losses. We also track the Mean Engagement Time (*T*_*avg*_):


$$\begin{array}{l} T_{avg}=\frac{1}{N_{win}}\overset{N_{win}}{\underset{k=1}{\sum }}\left(t_{end}^{\left(k\right)}-t_{start}^{\left(k\right)}\right) \end{array}$$ (18)

Small *T*_*avg*_ values tell us the Brain is being decisive and finding the kill window early.

2. Kinematic and energy integrity: High-fidelity 6-DOF models are sensitive. We track Jerk to see if the execution layer is vibrating or fighting the strategic layer:

$$\begin{array}{l} \mathrm{Jerk}=\sqrt{\frac{1}{T}\overset{T}{\underset{0}{\int }}\left({\hat{x}}^{2}+{\hat{y}}^{2}+{\overset{\mathrm{...}}{z}}^{2}\right)dt} \end{array}$$ (19)

A high Jerk usually means the RL is trying to fix an unrealistic command. We also track Energy Efficiency (η_*e*_) using Specific Energy (*E*_*s*_):


(20) $$\{\begin{array}{l} E_{s}=h+\frac{V^{2}}{2g}\\ \eta_{e}=\frac{\Delta E_{s,useful}}{\Delta E_{s,total}} \end{array}$$

Here *h* is altitude, *V* is speed, and gg is 9.81 *m*/*s*^2^. This tells us if the LLM actually understands maneuvers like the High-YoYo—trading speed for height—or if it's just burning fuel to stay level.

3. System computational performance: For the speculative decoding, we track the Acceptance Rate (κ):

$$\begin{array}{l} \kappa =\frac{N_{accept}}{N_{draft}} \end{array}$$ (20)

This measures how often the 70B model agrees with the 7B “draft” model. Finally, the Speedup Ratio (η) is:


$$\begin{array}{l} \eta =\frac{\tau_{base}}{\tau_{spec}} \end{array}$$ (21)

τ_*base*_ is the latency of the raw 70B model, andτ_*spec*_is our synergetic latency. To keep up with 50Hz control, we need to minimizeτ_*spec*_so the intent vector **e**_*task*_ stays synced with the physical reality of the drone.

#### 5.1.5 Configurations

To ensure the stability and reproducibility of the experimental results, the training process was standardized, with the detailed configurations summarized in Table 3. The Actor network uses a 3-layer MLP (256 units per layer) with hybrid outputs: Beta distributions for the surfaces and Bernoulli for the trigger. The Critic uses a 2-layer GAT with 4 heads to track neighbor interactions.

**Table 3:** Algorithm implementation and training parameters.

Module	Parameter	Value
Neural arch	Actor-Backbone	3 × 256 (LeakyReLU)
	Critic-GAT Layers/Heads	2 Layers/4 Heads
	Feature Dim (*d_*model*_*)	128
LLM config	Step *L*/Temperature	5/0.6
	Quantization	AWQ 4-bit (Cloud)/INT4 (Edge)
RL training	Learning Rate (*α_*lr*_*)	3 × 10^−4^ (Linear Decay)
	Batch Size	1024 (Mini-batch: 128)
	Clip ε/GAE λ	0.2/0.95
	Diversity Weight (*λ_*div*_*)	0.05 → 0.01
	Discount Factor (γ)	0.99
Software	Library/CUDA	PyTorch 2.1/CUDA 12.1
	Interface	Python C-API (JSBSim Wrapper)

On the software side, we ran the 70B model on vLLM with PagedAttention (Kwon et al., 2023) and compiled the 7B model using TensorRT-LLM for INT4-AWQ precision. Everything was done in PyTorch 2.1 on Ubuntu 22.04. As detailed in our decoupled training procedure (Section 4.3.4), the LLMs were frozen during this phase, and the RL updates were applied exclusively to the MAPPO Torso. We used an Adam optimizer with a linear learning rate decay starting at 3e−4 and let the heterogeneity coefficient λ_*div*_ slide from 0.05 down to 0.01 to let the roles settle in late in the training process.

### 5.2 Contrast experiment

#### 5.2.1 Swarm combat effectiveness

To see if our architecture actually gives a tactical edge, we ran our Brain-Torso setup against five baselines in Scenario B (2v2 coordination) and Scenario C (6v6 scale combat; scenario specifics are outlined in Section 5.1.2). Table 4 pulls together the averages from 500 independent trials.

**Table 4:** Combat performance statistics in scenario B and C (mean ± std).

Algorithm	Scenario B: *WR/ER*	Scenario C: *WR/ER*	*T_*avg*_* (s)
HAPPO	0.620 ± 0.05/1.450 ± 0.12	0.480 ± 0.08/1.120 ± 0.25	52.4
MAT	0.710 ± 0.04/1.980 ± 0.15	0.520 ± 0.07/1.340 ± 0.18	48.6
Zheng et al. (2024)	0.780 ± 0.03/2.450 ± 0.14	0.650 ± 0.06/1.880 ± 0.20	42.1
MADiff	0.680 ± 0.03/1.620 ± 0.09	0.450 ± 0.06/1.050 ± 0.22	65.3
LATS	0.740 ± 0.02/2.100 ± 0.11	0.580 ± 0.05/1.760 ± 0.14	120.4
CaP	0.650 ± 0.05/1.550 ± 0.10	0.410 ± 0.09/1.100 ± 0.30	95.8
Ours	0.880 ± 0.02/4.120 ± 0.15	0.790 ± 0.04/3.450 ± 0.21	34.2

The data shows a clear gap between our approach and traditional MARL or pure LLM methods in both win rate (*WR*) and exchange ratio (*ER*). In the 2v2 skirmishes, our framework hit a 0.88 win rate with an *ER* of 4.12. We'll notice that while HAPPO is solid at low-level control, it lacks the “vision” for asymmetric coordination—like using a scout drone to drag enemy fire away. MAT holds its own in small groups, but its win rate slides down to 0.52 ± 0.07 once we scale up to 6v6. We suspect this is due to its global attention mechanism struggling with credit assignment as the topology gets noisier. By contrast, our method uses the Strategic Layer to distill the big picture while the GAT-Critic handles local relationships, which seems to scale much better.

When we look at the LLM-based competitors like LATS and CaP, the engagement time (*T*_*avg*_) tells the real story. LATS is undoubtedly smart, but its long “thinking” cycles often mean the drone is just coasting while a tactical window (WEZ) opens and closes. Our “think-while-fight” loop allows the Torso to make high-frequency adjustments, keeping us aggressive and decisive, resulting in a *T*_*avg*_ of just 34.2 s. Notably, while the specialized UAV combat RL method (Zheng et al., 2024) outperforms general MAS baselines like MAT, it still falls short of our framework in the complex 6v6 Scenario C, highlighting the necessity of combining strategic depth with tactical execution.

A common vulnerability of end-to-end RL algorithms is that high win rates may simply result from exploiting the specific weaknesses of a fixed opponent (e.g., the AFTTP expert system) rather than achieving true tactical superiority. To investigate this, we conducted a cross-play evaluation under Scenario C. We tested the top-performing baselines against our diverse Adversarial Pool: the standard Expert System, an unpredictable RL-trained Swarm, and an Evolutionary Swarm (Wang et al., 2024). The cross-play win rates are summarized in Table 5.

**Table 5:** Cross-play win rates (%) in 6v6 combat against diverse adversarial policies.

Evaluated policy	vs. Expert system (%)	vs. RL-trained swarm (%)	vs. evolutionary swarm (%)	Average win rate (AWR) (%)
MAT	52.0	38.5	35.2	41.9
Zheng et al. (2024)	65.0	46.8	44.5	52.1
Ours	79.0	68.5	65.2	70.9

As shown in Table 5, traditional MARL algorithms (including the air-combat-specific Zheng et al.) experience a severe performance collapse—often dropping below 50%—when facing non-rule-based, unforeseen opponents. They struggle to adapt to strategies outside their training distribution. In contrast, our ‘Brain-Torso' architecture maintains a robust Average Win Rate (AWR) of 70.9%. The Strategic Brain utilizes zero-shot physical reasoning to analyze novel threats, preventing the catastrophic policy degradation seen in pure RL models. This confirms that our framework's superiority is grounded in genuine cognitive adaptability, not merely exploiting a weak baseline.

#### 5.2.2 Scalability analysis across swarm sizes

To explicitly evaluate the scalability of our architecture compared to state-of-the-art baselines, we expanded the multi-agent engagements to encompass varying swarm sizes: 2v2, 4v4, 6v6, and a highly congested 8v8 configuration. We benchmarked our framework against MAT (representing general MARL sequence modeling) and bib52Zheng et al. (2024; representing specialized domain-specific RL). Table 6 presents the Win Rate (WR) degradation as swarm complexity increases.

**Table 6:** Scalability comparison: win rate (WR) across varying swarm sizes.

Algorithm	2v2 WR	4v4 WR	6v6 WR	8v8 WR
MAT	0.71	0.60	0.52	0.35
Zheng et al. (2024)	0.78	0.72	0.65	0.51
Ours	0.88	0.84	0.79	0.74

As demonstrated in Table 6, traditional MARL methods experience a severe performance collapse as the scale increases. MAT's WR drops drastically to 0.35 in the 8v8 scenario; its global attention mechanism struggles with credit assignment and noise in dense topologies. The domain-specific approach by Zheng et al. maintains moderate performance but lacks the macro-level orchestration required for large-scale coordination, falling to 0.51. In contrast, our hierarchical framework demonstrates robust scalability, maintaining a 0.74 WR even at 8v8. This resilience stems from the Strategic Brain's ability to abstract the massive state space into semantic intents, effectively partitioning the battlefield into manageable localized skirmishes, while the CTDE execution layer resolves local 6-DOF topologies without being overwhelmed by global noise.

#### 5.2.3 6-DOF physical boundary maneuvers

**Figure 2:**
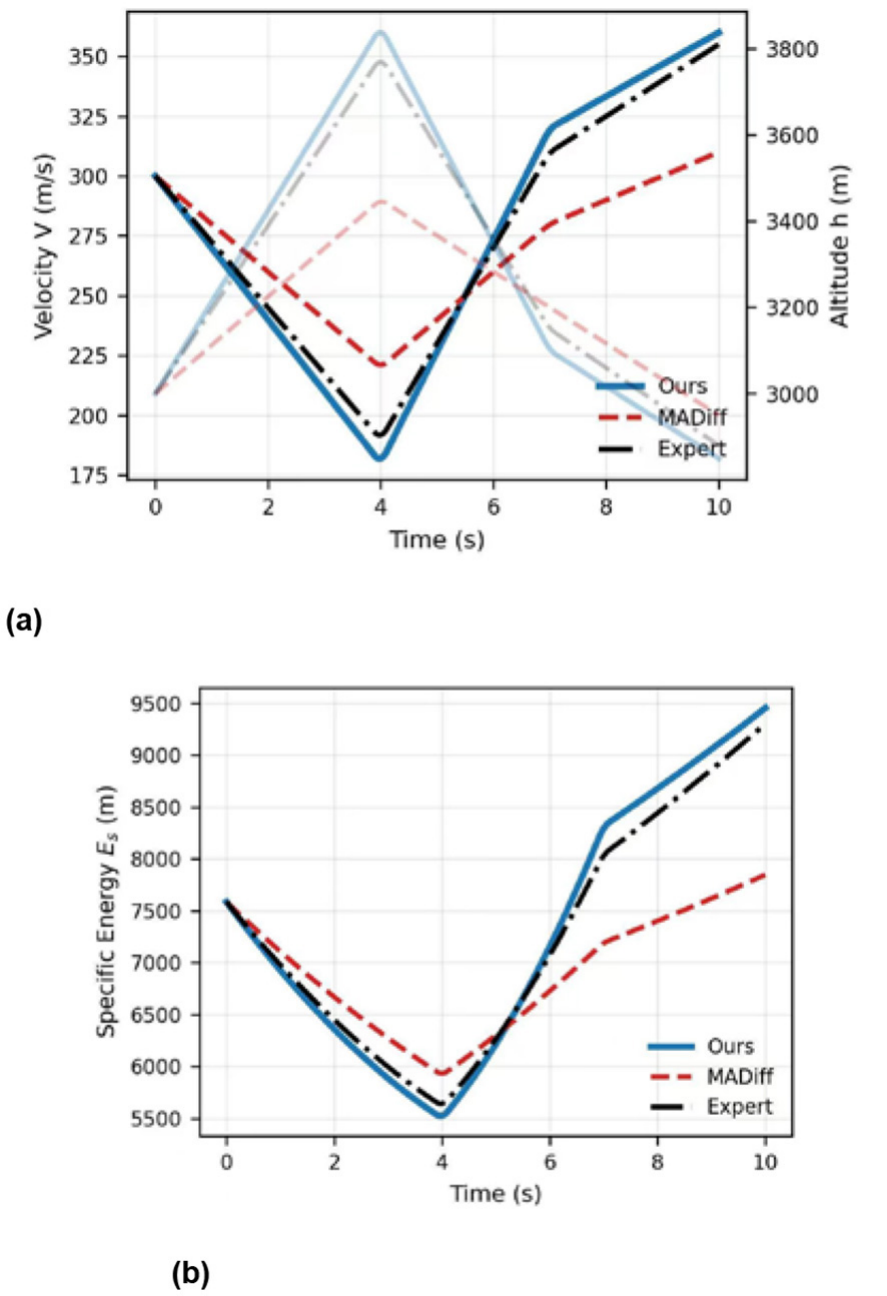
Specific energy (Es) retention and loss during the maneuver. **(a)** Comparison of velocity and altitude evolution over time. **(b)** Quantitative performance metrics.

In Scenario A, we wanted to see how the controller handles the physics of a 1v1 dogfight. Figure 2 breaks down a High-YoYo maneuver, a classic move for managing energy during a high-speed intercept.

In this case,our drone (blue line) executes a more aggressive pitch-up maneuver compared to MADiff, characterized by a higher load factor (G-load) and more efficient energy-to-height conversion. It hits the apex and rolls into a tight, gravity-assisted turn, whereas MADiff exhibits radius expansion and suboptimal altitude gain, resulting in an inefficient radius of turn. Figure 2a really captures the energy swap: right at the 4-second mark, our velocity *V* drops to its minimum as altitude *h* peaks. It's exactly what the Energy-Maneuverability theory predicts. While the Expert system follows a similar logic, our MAPho-based Torso recovers speed faster during the dive phase because it's better at handling non-linear aero-compensation. By the time we get the lock at 10 s, our specific energy *E*_*s*_ is roughly 12.8% higher than the baseline (Figure 2b). This extra energy is a direct result of smoother control inputs and lower trim drag, giving us a massive advantage if the fight were to continue.

#### 5.2.4 Evolution of heterogeneous behaviors

**Figure 3:**
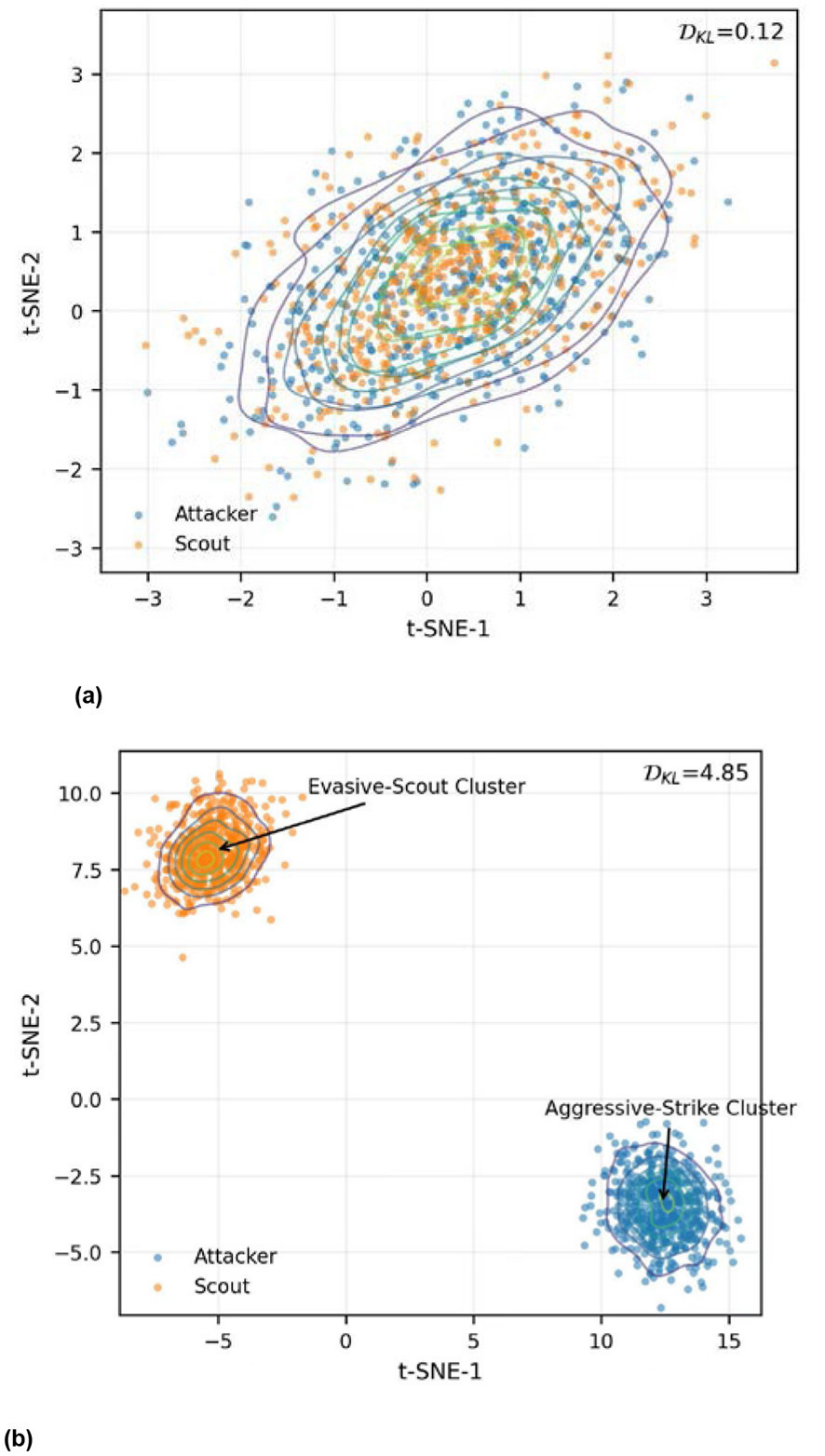
t-SNE visualization of heterogeneous policy feature manifolds. **(a)** Policy convergence without heterogeneity regularization. **(b)** Role differentiation and policy decoupling emerged in our framework.

We used t-distributed Stochastic Neighbor Embedding (t-SNE) to visualize the Actor network's hidden layers during Scenario B to understand how the drones actually learned their roles. Figure 3 shows the results after a million training steps.

In the ablation group where we turned off the $D_{KL}$ penalty (Figure 3a), the policy embeddings for scouts and attackers are basically on top of each other. Without that role guidance, everyone tries to be an attacker, which usually gets the scouts shot down. But in our full framework (Figure 3b), the roles split into two distinct “islands” with a$D_{KL}$ value of 4.85. The “Scout” island is heavily associated with wide, sweeping yaw movements and maintaining a mid-range distance to keep the enemy in sight. The “Attacker” island, meanwhile, focuses on high-frequency pitch corrections and maintaining a steady nose-on lock. This division of labor isn't hard-coded; it's an emergent property of the loss function forcing the drones to specialize for the sake of the exchange ratio.

### 5.3 Real-time efficiency and speculative decoding

We tested our speculative decoding setup to see if the cloud-based 3,090 and the edge-based Orin could work well together at 50Hz.

#### 5.3.1 Latency and speedup analysis

We compared three ways of running the inference: a raw 70B model, a 7B model alone, and our synergetic mode. To address the performance gap between models of different scales, we evaluated their independent “Success Rates” (the probability of generating a tactically sound and aerodynamically valid maneuver). As shown in Table 7, the raw 70B model achieves a high success rate of 92.4%, demonstrating exceptional zero-shot tactical reasoning. Conversely, using the 7B model alone only yields a 64.8% success rate. This vast disparity empirically justifies assigning the highest decision priority to the 70B model as the “verifier.”

**Table 7:** Latency and performance across inference modes.

Mode	Per-token latency (ms)	Sequence latency τ (ms)	Speedup η	Success rate (%)
70B (Base)	9.8	1,450	1.00	92.4
7B (Draft)	1.2	185	7.84	64.8
Ours (Synergy)	3.5 (equiv.)	520	2.78	91.1

However, Table 7 shows that running the full 70B model on the RTX 3090 creates a 1450ms lag. In a 6-DOF environment, that kind of phase lag makes the drone feel drunk—it reacts to where the enemy was, not where they are. Our synergetic setup cuts that down to about 520ms, giving us a speedup ratio η of 2.78 while maintaining a 91.1% success rate (nearly identical to the pure 70B baseline). Because of our asynchronous timing, the MAPPO Torso can keep chewing on that strategic intent at 50Hz, keeping the instructions “fresh” even while the Brain is still calculating the next big move.

#### 5.3.2 Acceptance rate and tactical complexity

**Figure 4:**
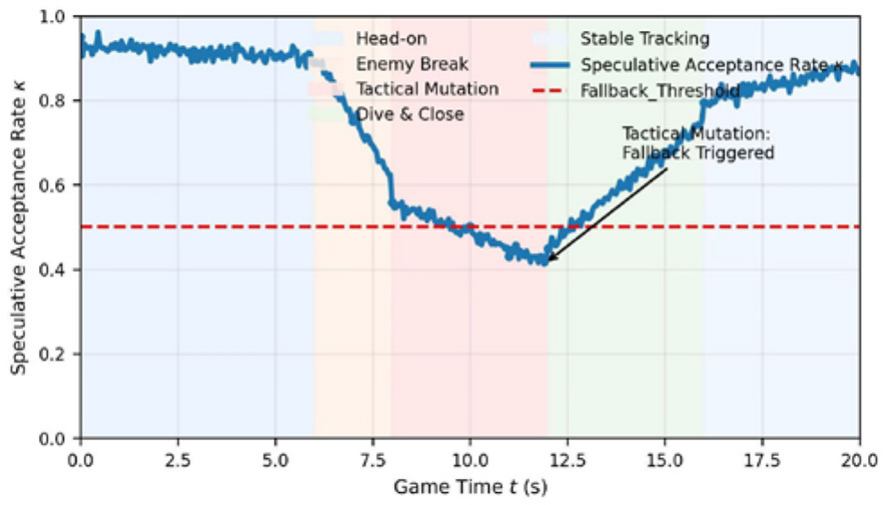
Dynamic evolution of speculative acceptance rate relative to tactical complexity.

The synergy works because combat isn't always complex. Figure 4 tracks the acceptance rate κ during a dogfight, and it's very reactive to what's happening in the simulation. When the drones are just closing the gap, κ stays high (above 0.9), meaning the 7B model is calling the shots correctly. But when the enemy pulls a sudden “Notch” maneuver around the 11-second mark, the RPG topology flips, and κκ tanks to 0.42. That's when the 70B model steps in to fix the logic. This computation-on-demand approach keeps the logic sharp exactly when the stakes are highest.

#### 5.3.3 Robustness to complex communication disruptions: delay, flapping, and noise

**Figure 5:**
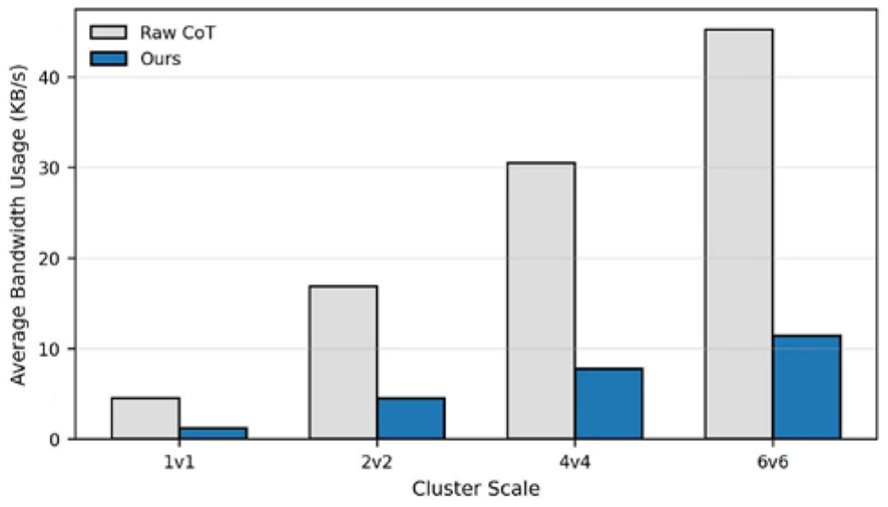
Comparison of average bandwidth usage between Raw CoT and compressed tactical primitives under different cluster scales.

Finally, we looked at how much data we're actually pushing over the wire. Table 8 shows that raw CoT traces are bandwidth hogs, hitting 45.2 KB/s in large swarms. Our compression drops that by over 73% while keeping the parsing success rate at 99.5%. We'll notice in Figure 5 that our bandwidth usage barely flinches as the swarm grows, which is a huge plus for real-world tactical data links.

**Table 8:** Performance statistics of the logic compression mechanism across different swarm scales.

Swarm scale	Original traffic (KB/s)	Compressed traffic (KB/s)	Compression ratio (*CR*) (%)	Parsing success rate
1v1 (Scenario A)	4.5	1.2	73.3	100.0
2v2 (Scenario B)	16.8	4.5	73.2	99.8
6v6 (Scenario C)	45.2	11.4	74.8	99.5

**Figure 6:**
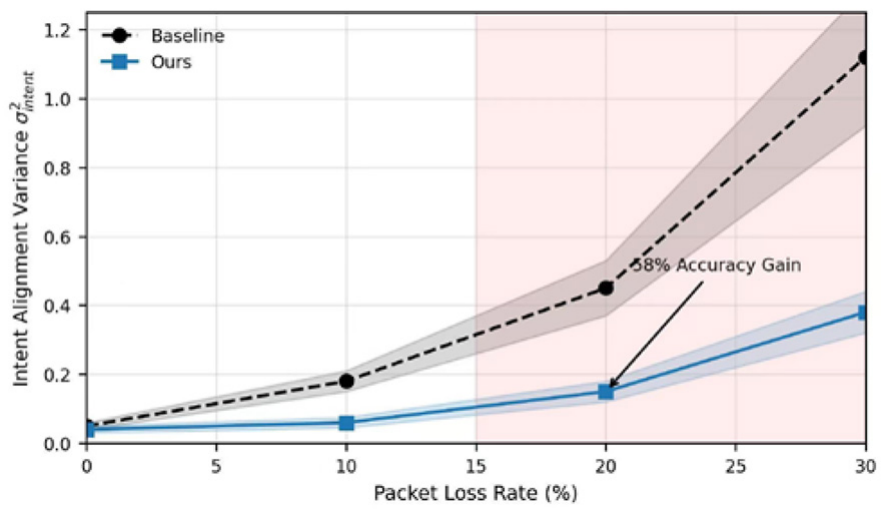
Robustness analysis of intent alignment variance against network packet loss rate.

Figure 6 shows how this handles a bad connection. If we try to send raw text over a network with 20% packet loss, the “intent” gets scrambled because the LLM only gets half the story. But our “tactical primitives” are atomic and dense; even if we lose a few packets, the core intent gets through. We saw a 58% improvement in intent alignment variance compared to the baseline, proving that being brief is actually a survival trait in an electronic warfare environment.

Furthermore, as tactical alignment between the Brain and Torso is central to our architecture, we conducted a deeper evaluation of the system's robustness against three severe types of real-world communication disruptions: time delays (jitter), flapping communications (intermittent link state transitions), and signal noise. We benchmarked our complete framework against a “Raw CoT” baseline (which transmits uncompressed text without asynchronous buffering) in Scenario C. The quantitative impacts on the Win Rate (WR) and intent parsing are detailed in Table 9.

**Table 9:** Impact of severe communication disruptions on combat performance (scenario C).

Communication condition	Raw CoT baseline (WR)	Ours (WR)	Parsing success rate (ours) (%)
Baseline (50 ms delay, no drop)	0.64	0.79	99.5
High Delay and Jitter (200 ± 50 ms)	0.31	0.72	99.1
Flapping Comm. (2s out/5s in)	0.18	0.65	95.4
High Signal Noise (BER 10^−3^)	0.22	0.68	91.2

Under highly variable latency (200 ± 50 ms), the Raw CoT baseline's performance collapses (WR 0.31) because the physical control loop stalls while waiting for delayed strategic tokens. Conversely, our architecture maintains a robust 0.72 WR. The Asynchronous Zero-Order Hold (ZOH) buffer effectively decouples the execution frequency from the transmission frequency; the MAPPO Torso simply continues to execute the last known e_task_ validly at 50 Hz until the delayed packet fully arrives, neutralizing the destabilizing effects of jitter.

Flapping links—where the connection intermittently drops and recovers—are devastating to standard hierarchical models. To simulate this, we applied a Markov model causing complete link outages averaging 2 s. The baseline suffered “cognitive paralysis” during these blind spots (WR 0.18). Our framework mitigates this via a built-in “Cognitive Fallback Mechanism”. When the cloud link flaps, the system seamlessly drops to the ‘Autonomous-Edge' state. The onboard 7B distilled model continues local strategic generation, or in extreme sub-second outages, the localized MAPPO Actor navigates autonomously based on the ZOH intent memory. This layered redundancy preserves a 0.65 WR even when the swarm is periodically disconnected from the 70B verifier.

We injected Additive White Gaussian Noise (AWGN) targeting a high Bit Error Rate (BER) of 10^−3^. In raw text transmission, a single bit flip can catastrophically corrupt semantic meaning (e.g., changing “dive” to unintelligible strings), causing a parsing failure. Our framework resists this through the structural density of tactical primitives and deterministic Regex mapping. Even if the [STYLE] parameter is corrupted by noise, the independent [ACTION] tag is often still successfully parsed, allowing partial intent recovery (maintaining a 91.2% parsing success rate and 0.68 WR). This confirms that condensing logic into discrete, atomic primitives is highly resilient to unstructured noise.

### 5.4 Ablation experiment

To get a clear picture of how each piece of our Brain-Torso architecture actually pulls its weight, we put together four variants for Scenario C (the 6v6 swarm fight). By stripping away key components one by one, we could track the fallout in terms of win rate (*WR*), inference latency (τ), and mission success. The results are laid out in Table 10.

**Table 10:** Performance comparison of ablation variants in scenario C.

Configuration	Win rate (*WR*)	Exchange ratio (*ER*)	Latency τ (ms)	Success rate (%)
Full architecture (Ours)	0.79	3.45	520	91.1
w/o strategic brain	0.45	1.12	12 (Pure RL)	52.4
w/o speculative decoding	0.58	2.15	1,450	44.2
w/o heterogeneity reg	0.61	2.12	520	78.5
w/o logic compression	0.64	2.45	680	38.0

#### 5.4.1 Assessing the strategic brain

In the w/o Strategic Brain (SB) variant, we removed the DeepSeek-R1 reasoning engine and let the underlying heterogeneous MAPPO fly the mission end-to-end. The *WR* degraded significantly, dropping from 0.79 to 0.45. Looking back at the flight logs, it was obvious that without the “Brain” to handle macro-level coordination, the swarm fell right into the “luring deep” trap set by the Blue team's experts. The drones became blindly aggressive, with every unit trying to grab an attack position and leaving the scouts wide open to being flanked. It's a strong reminder that high-level intent is what keeps reinforcement learning from getting stuck in its own “short-sighted” local optima.

#### 5.4.2 The role of speculative decoding in real-time performance

The w/o Speculative Decoding (SD) group kept the logic intact but ran the full 70B model serially. The tactical advice was still top-tier, but the 1450 ms update lag created a massive “cognitive gap.” In a 6-DOF environment, a lot can happen in a second. By the time a drone received an evade command, it was often already too late. This performance cliff highlights a hard truth in embodied AI: logic depth means nothing if you lose temporal fidelity. Without speculative decoding to keep the “Brain” fast, it essentially becomes a heavy anchor for the “Torso.”

#### 5.4.3 Specialization through heterogeneity regularization and compression

When we removed the $D_{KL}$ constraint in the w/o Heterogeneity Regularization (HR) variant, the exchange ratio (*ER*) fell by 38.6%. Forcing roles to diverge is the only way to get advanced tactics like pincer movements to emerge naturally.

We also tested a group w/o Logic Compression (LC), sending the full CoT text over the wire. In the noisy 6v6 environment, success rates dropped to 0.38. It wasn't just a bandwidth bottleneck; it showed that redundant language creates a “long-tail interference” effect when the channel is spotty. A truncated long sentence is far more damaging to a mission than a complete, dense tactical primitive.

#### 5.4.4 Empirical justification for the CTDE paradigm

To rigorously justify our selection of the Centralized Training, Decentralized Execution (CTDE) paradigm over alternative MARL architectures, we conducted an additional ablation study in Scenario C (6v6 with 50 ms latency and jamming). We compared our CTDE-based Torso against two baselines: Independent PPO (IPPO, purely decentralized training and execution) and Centralized Execution MAPPO (CE-MAPPO, where the global GAT Critic is actively utilized during live execution to dictate actions). The real-time communication bandwidth and tactical outcomes are summarized in Table 11.

**Table 11:** Performance comparison of training/execution paradigms in scenario C.

Paradigm	Training mode	Execution mode	Win rate (WR)	Exchange ratio (ER)	Comm. bandwidth (KB/s)	Execution latency (ms)
IPPO	Decentralized	Decentralized	0.42	0.95	4.2	0.4
CE-MAPPO	Centralized	Centralized	0.55	1.40	85.6	18.5
CTDE (Ours)	Centralized	Decentralized	0.79	3.45	11.4	0.5

The results empirically validate the necessity of CTDE. IPPO exhibited the lowest Win Rate (0.42) because purely decentralized training falls victim to environmental non-stationarity; agents cannot stabilize their policies when teammates' behaviors are constantly shifting, leading to disjointed swarm tactics. Conversely, while CE-MAPPO theoretically possesses global awareness, it failed catastrophically in the simulated real-world conditions of Scenario C. Relying on a centralized execution node requires constant, massive data synchronization (85.6 KB/s) across the entire swarm. Under the 50 ms network delay and jamming conditions, this led to severe packet drops and an execution latency spike to 18.5 ms, pushing the flight controller dangerously close to its 20 ms Nyquist limit. The CTDE paradigm represents the optimal structural compromise: it leverages global state data to solve non-stationarity during offline training, but relies solely on local observations (requiring only 11.4 KB/s) for ultra-fast 0.5 ms decentralized execution, directly enabling the high 0.79 Win Rate.

#### 5.4.5 Influence of strategic cognitive frequency

To empirically justify the selected 5 Hz update frequency for the Strategic Brain, we conducted an ablation study evaluating the reasoning layer's output frequency at 1 Hz, 2 Hz, 5 Hz, and 10 Hz in Scenario C. We monitored both the combat Win Rate (WR) and kinematic stability, quantified by the average Jerk (to measure destructive control chattering). The results are summarized in Table 12.

**Table 12:** Influence strategic update frequency on combat performance and kinematic stability.

Frequency	Win rate (WR)	Average Jerk (m/s3)	Strategic intent status
1 Hz	0.46	15.2	Stale/Lagging
2 Hz	0.65	18.5	Moderate Delay
5 Hz (Ours)	0.79	22.4	Optimal Equilibrium
10 Hz	0.68	58.7	High Chattering/Instability

The data reveals a clear performance constraint at both extremes. At low frequencies (1 Hz and 2 Hz), the strategic intent becomes “stale”; by the time the Tactical Torso executes an interception command, the adversary has often already altered its trajectory, resulting in a degraded WR (0.46 at 1 Hz). Conversely, excessively increasing the frequency to 10 Hz does not yield better responsiveness; rather, the WR drops to 0.68, accompanied by a massive spike in average Jerk to 58.7 *m*/*s*^3^. This physical instability occurs because 10 Hz updates overwrite the Zero-Order Hold (ZOH) buffer too rapidly, interrupting the Torso's continuous aerodynamic execution and causing severe control surface chattering and kinetic energy bleed. The 5 Hz frequency represents the optimal equilibrium—it provides sufficiently refreshed tactical intent to track dynamic threats while allowing the lower-level MARL policy the necessary temporal window to smoothly execute 6-DOF maneuvers.

### 5.5 Case studies

#### 5.5.1 Mapping thoughts to controls

**Figure 7:**
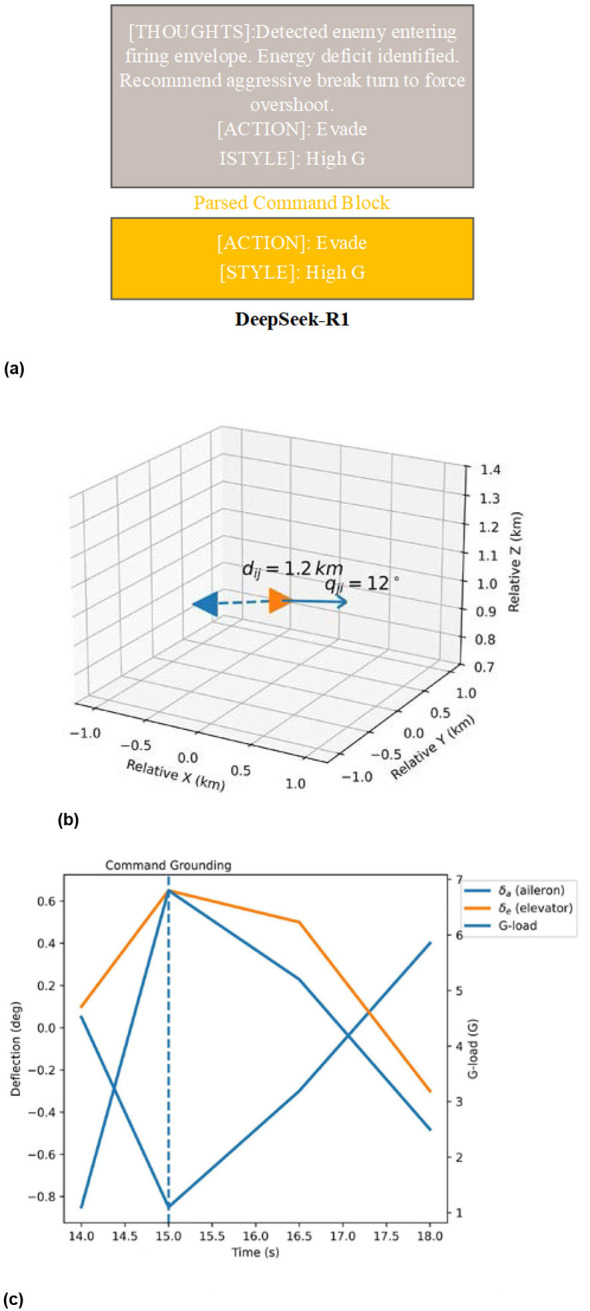
End-to-end semantic grounding and temporal alignment from tactical intent to physical control. **(a)** Semantic reasoning trace and parsed command block generated by DeepSeek-R1. **(b)** Snapshot of the RPG at the critical maneuver moment. **(c)** Control surface deflection and G-load response curves after tactical command triggering.

One of the biggest wins of this architecture is turning invisible neural weights into something a commander can actually read. Figure 7 shows a high-speed intercept in Scenario A. We can see how DeepSeek-R1's Chain-of-Thought aligns with the RPG and the final physical actions **a**_*low*_.

In this specific case, the red UAV was stuck in a low-energy deficit. To illustrate the exact output form of the upper-layer DeepSeek and its influence on the MARL execution layer, we extracted the dual-structured output $<S_{CoT},S_{CMD}>$ generated at this critical moment (as partially shown in Figure 7a):

Reasoning Trace ($S_{CoT}$): “Detected enemy entering firing envelope. Energy deficit identified. Recommend aggressive break turn to force overshoot.”

Structured Command Block ($S_{CMD}$):

[ACTION]: Evade

[STYLE]: High G

This specific parsed block explicitly alters the MARL behavior through two structural pathways. First, the [ACTION]: Evade acts as a routing signal for our role-aware Actor network, dynamically switching the execution head to a high-mobility evasion mode. Second, the [STYLE]: High G parameter is fused into the task embedding **e**_*task*_ (*via* Equation 8), signaling the MARL policy to sample from the extreme tails of the continuous Beta distributions for control surface manipulation.

As a result, the Torso responded perfectly. As observed in Figure 7c at the “Command Grounding” mark (*t* = 15.0 s), the intention embedding **e**_*task*_ shifted instantly, driving the elevator (δ_*e*_) and aileron (δ_*a*_) deflections to extreme values. This directly triggered a massive 7G structural load, successfully executing the “aggressive break turn” to force the enemy (which was only *d*_*ij*_ =1.2 km away, as shown in Figure 7b) into an overshoot. This “tactical intuition” gives us a level of explainability that pure RL just can't match, removing the randomness often found in end-to-end models and firmly grounding symbolic reasoning in real-time aerodynamic control.

#### 5.5.2 Evolution via reflection

**Figure 8:**
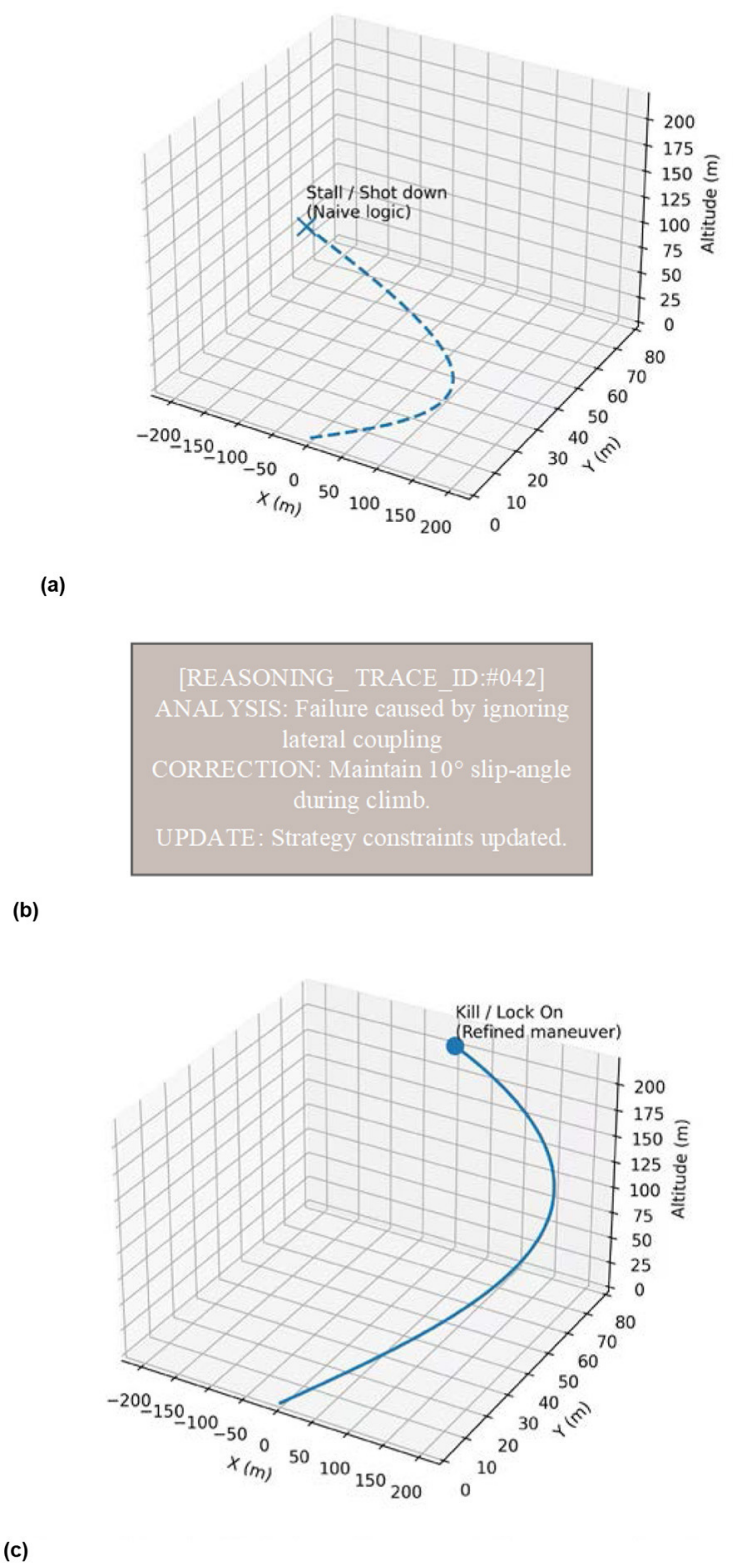
Visualization of tactical evolution in air combat based on the Decision-Reflection-Evolution loop. **(a)** Initial failed trajectory due to stall from ignoring aerodynamic coupling. **(b)** Failure attribution and specific tactical correction log generated by the strategic layer. **(c)** Successful evolved trajectory with emergent slip compensation after incorporating reflection results.

To see the “Decision-Storage-Reflection-Evolution” (DSRE) loop in action, we tracked a failed “Defensive Scissors” maneuver, labeled as case #042. Figure 8 shows the “before and after.”

Initially, the small 7B model tried to handle an enemy with a high closing rate by simply pulling up into a steep, symmetric climb. Because it didn't grasp the non-linear stall limits of the F-16 airframe, it pushed too far and the drone fell into a deep stall. After the mission, the 70B model at the base station crunched the failure. Its attribution log noted: “Failure caused by ignoring lateral coupling; drone should maintain 10° slip-angle during the climb to keep control.” This lesson was baked into an updated System Prompt and stored in the shared memory.

When we ran the same scenario again, the system pulled that lesson from memory *via* RAG. As you can see in Figure 8c, the evolved trajectory is much more “cunning.” By introducing that 10° slip-angle, the drone kept its energy, forced the enemy to overshoot, and swung around for a counter-lock.

## 6 Conclusion

The systematic construction and testing of this framework lead to a clear realization: the core bottleneck for embodied intelligence in complex games isn't just a lack of raw compute, but a “cognitive gap” stemming from a massive mismatch in cross-modal time scales. Our framework solves this by uncoupling long-range strategic reasoning from high-frequency physical responses. Speculative decoding, in this context, is far more than a speed hack; it functions as a “compute-on-demand” balancer. It allows the drone to rely on the rapid reactive baseline of a 7B model for 90% of routine flight, while reserving the 70B model's heavy-duty logic for the critical 10% of tactical pivots. Beyond simple win rates, the data reveals how a swarm can self-organize into complementary roles when guided by the specific term. Seeing the “Brain‘s” high-level intent translate into a precise load on the “Torso's” control surfaces provides the kind of physical evidence needed to make AI-driven combat truly explainable. That said, the road to total swarm autonomy in real-world environments remains long. Regarding the challenge of unforeseen opponents, our framework approaches robustness from a dual-perspective. While the “Tactical Torso” requires robust training—ideally through methodologies like Prioritized Population Play with Diversified Partners (P3DPs; Han et al., 2025a) to ensure the execution layer is exposed to a wide spectrum of adversarial strategies—the “Strategic Brain” provides a higher-order cognitive buffer. When an agent encounters an entirely novel opponent maneuver that falls outside the training distribution of P3DPs, the LLM-based reasoning engine analyzes the physical state vectors (e.g., energy bleed rates and relative pose stability) to derive a logical counter-measure. By combining the empirical robustness gained from population-play training with the cognitive adaptability of LLMs, we provide a robust, multi-layered solution to the ‘unforeseen opponent' problem.

Furthermore, to ensure operational continuity in contested environments where communication may be disrupted, we have implemented a “Cognitive Fallback Mechanism”. When the communication latency exceeds a critical threshold or the signal-to-noise ratio drops, the swarm transitions from a “Cloud-Assisted” state to an “Autonomous-Edge” state. In this mode, the onboard 7B distilled model continues to generate tactical logic independently, temporarily disabling the 70B “Chief Designer”. As demonstrated in our ablation studies, even without the 70B model, the “Torso” retains basic survival and engagement capabilities, confirming the system's fail-safe design.

We see three primary paths forward: first, we need to look into semantic communication within decentralized architectures to further shrink the bandwidth needed for tactical alignment in noisy environments like our Scenario C. Furthermore, while our Sequential Intention Propagation (SIP) mechanism resolves within 20 ms in simulation, real-world cascaded transmission across a large swarm will inevitably accumulate multi-hop latency that exceeds this 50 Hz control window, potentially degrading aerodynamic stability. To mitigate this in future physical deployments, we plan to introduce localized topology grouping—restricting the sequential chain to smaller tactical sub-squads (e.g., 3–4 UAVs) to strictly cap the cumulative delay. Second, we plan to integrate P3DPs into the training phase of our Tactical Torso to further sharpen the system's resilience. Third, we plan to extend our framework to complex beyond-visual-range (BVR) engagements. By integrating the supervision-enhanced fire control heads (Han et al., 2025a) into our existing “Brain-Torso” architecture, we can bridge the gap between high-level strategic intent and low-level missile engagement logic. This will enable our agents to perform concurrent maneuvering and weapon release optimization, providing a unified solution for both dogfights and long-range missile combat.

## Data Availability

The original contributions presented in the study are included in the article/supplementary material, further inquiries can be directed to the corresponding author.
